# *Escherichia coli* coculture for de novo production of esters derived of methyl-branched alcohols and multi-methyl branched fatty acids

**DOI:** 10.1186/s12934-022-01737-0

**Published:** 2022-01-15

**Authors:** Fernando Bracalente, Martín Sabatini, Ana Arabolaza, Hugo Gramajo

**Affiliations:** Microbiology Division, Facultad de Ciencias Bioquímicas Y Farmacéuticas, IBR (Instituto de Biología Molecular Y Celular de Rosario), Consejo Nacional de Investigaciones Científicas Y Técnicas, Universidad Nacional de Rosario, Ocampo y Esmeralda, 2000 Rosario, Argentina

**Keywords:** *E. coli* coculture, Multi-methyl-branched fatty acids, Methyl-branched alcohols, Multi-methyl-branched esters, Fed-batch culture, Response surface methodology

## Abstract

**Background:**

A broad diversity of natural and non-natural esters have now been made in bacteria, and in other microorganisms, as a result of original metabolic engineering approaches. However, the fact that the properties of these molecules, and therefore their applications, are largely defined by the structural features of the fatty acid and alcohol moieties, has driven a persistent interest in generating novel structures of these chemicals.

**Results:**

In this research, we engineered *Escherichia coli* to synthesize de novo esters composed of multi-methyl-branched-chain fatty acids and short branched-chain alcohols (BCA), from glucose and propionate. A coculture engineering strategy was developed to avoid metabolic burden generated by the reconstitution of long heterologous biosynthetic pathways. The cocultures were composed of two independently optimized *E. coli* strains, one dedicated to efficiently achieve the biosynthesis and release of the BCA, and the other to synthesize the multi methyl-branched fatty acid and the corresponding multi-methyl-branched esters (MBE) as the final products. Response surface methodology, a cost-efficient multivariate statistical technique, was used to empirical model the BCA-derived MBE production landscape of the coculture and to optimize its productivity. Compared with the monoculture strategy, the utilization of the designed coculture improved the BCA-derived MBE production in 45%. Finally, the coculture was scaled up in a high-cell density fed-batch fermentation in a 2 L bioreactor by fine-tuning the inoculation ratio between the two engineered *E. coli* strains.

**Conclusion:**

Previous work revealed that esters containing multiple methyl branches in their molecule present favorable physicochemical properties which are superior to those of linear esters. Here, we have successfully engineered an *E. coli* strain to broaden the diversity of these molecules by incorporating methyl branches also in the alcohol moiety. The limited production of these esters by a monoculture was considerable improved by a design of a coculture system and its optimization using response surface methodology. The possibility to scale-up this process was confirmed in high-cell density fed-batch fermentations.

**Supplementary Information:**

The online version contains supplementary material available at 10.1186/s12934-022-01737-0.

## Background

Oleochemicals are a group of fatty acids (FA) derived compounds that have traditionally been derived from vegetable oils and animal fats via chemical or enzymatic processes [1, 2]. The diversity in chemical structures of these molecules (e.g., FA, alkanes, alcohols, esters) gives them a wide range of physicochemical properties, allowing the manufacture of different commercial products such as lubricants, biodiesel, detergents, printing inks, rubbers, adhesives, cosmetics and coating stuffs [3]. Although the oleochemical industry has grown considerably during the past years, the limited availability, sustainability, and high cost of feedstocks is limiting the continued growth of this sector [4]. During the last decade, biotechnology has emerged as an alternative process, where oleochemicals-like compounds can be produced using microbial catalysis. In this sense, the lipid metabolism of various industrial microorganisms has been engineered toward the production of a wide range of oleochemicals [3]. Particularly, a variety of alkyl oxo-esters, ranging from long- to medium-chain fatty acid-methyl- and -ethyl-esters (FAME and FAEE), as well as, mono-esters of long-chain fatty acids and fatty alcohols (wax esters) have been produced in *E. coli*. These developments were achieved combining general metabolic engineering strategies to maximize the flux through FA biosynthesis and using different ester producing enzymes (such as methyltransferases [5, 6], alcohol acetyltransferases [7] or wax ester synthases/diacylglycerol acyltransferases [8, 9]).

Furthermore, considering the numerous and highly relevant industrial applications of fatty esters and the fact that their physical properties and therefore their performance, are largely defined by the structural features of the FA and alcohol moieties [10], several new fatty esters not found in nature, were designed and produced in bacteria [11]. One of the most interesting examples are the compounds generated by Menéndez Bravo et al*.* [12], who took advantage of the in-depth genetic and biochemical understanding of FA synthesis in the model organism *E. coli* to engineer a mycocerosic acid (MA) polyketide synthase-based biosynthetic pathway from *Mycobacterium tuberculosis* and redefined its biological role towards the production of multi-methyl-branched esters (MBE). These MBE, with novel chemical structures and properties, could be used, for example, for the development of new and improved bio-based molecules with high added value (e.g., specialty chemicals) [12, 13]. The *E. coli* platform for the production of these compounds contained three heterologous enzymes of the MA pathway that enabled the biosynthesis of multi-methyl-branched-fatty acids (MBFA): (1) the acyl-AMP ligase FadD28, that loads the long-chain FA onto the mycocerosic acid polyketide synthase Mas, (2) the Mas enzyme that elongates the linear-chain FA loaded in this multidomain protein with four elongation cycles, using methylmalonyl-CoA as extender unit, and (3) the polyketide-associated protein A5 (PapA5) which catalyzes the release and transesterification of the MBFA with an alcohol. Based in the high flexibility of this heterologous biosynthetic pathway, this *E. coli* platform allowed the synthesis of a wide set of related ester molecules by feeding different alcohols and different long-chain FA to the culture medium [12].

A highly challenging aim in microbial production of advanced chemicals is the generation of an integrated bioprocesses in which product synthesis occurs directly through conversion of a simple carbon source [14]. In this context, and in order to circumvent the need of an exogenous alcohol supply, heterologous biosynthetic pathways for de novo production of the alcohol substrate have been engineered into *E. coli* to produce biofuels [8, 15]. This strategy has also been successfully applied for the production of MBE derived from long-chain carbon alcohols, like C_16_-C_18_ fatty alcohols [12], as well as from short-chain carbon alcohols, such as ethanol [16].

Considering that *E. coli* strains have been modified for the biosynthesis of branched-chain alcohols (BCA), like isobutanol and isopentanol (3-methyl 1-butanol) [17, 18], in this work we set out to generate an *E. coli-*based system that could produce MBE structures derived from the esterification of the MBFA and these alcohols, with the only addition of propionate to the culture. For this, and taking into account all the potential benefits of a modular coculture engineering approach, such as overcoming metabolic burden by division of labor, optimization of pathways in a modular fashion, and balancing the metabolic fluxes between individual modules by controlling the ratio of engineered strains [19, 20]; we also applied this novel strategy for the production of BCA-derived MBE. Therefore, in order to develop a bioprocess for de novo production of BCA-derived MBE, two different platforms were built and tested: 1) a unique strain containing both, the MA and the BCA pathways, and 2) a coculture system consisting in two different *E. coli* strains, one producing the BCA and the other generating the MBFA and the final MBE product.

## Results and discussion

### Biosynthesis of branched-chain alcohols derived MBE in E. coli

Feeding experiments for cultures of RQ5.0 strain (RQ5 harboring plasmid pMB07) with isobutanol or isopentanol, lead to the production of BCA-derived MBE at low yields (Additional file 2: Fig S1A). For improving MBE titers, as reported previously, the expression system for the *M. tuberculosis fadD28, mas* and *papA5* genes is clearly relevant, i.e. the inducible promoter used for controlling their expression and the copy number of the vector that harbors these genes [13]. In this sense, plasmid pMB07, constructed to contain *fadD28*, *mas* and *papA5* genes cloned in an operon configuration downstream the T7 promoter in a pBR322 derivative vector, was selected as the starting expression system [13]. In this work, and in order to continue the optimization process (specifically the BCA-derived MBE yields) we constructed pMB24, a new plasmid in which we kept the origin of replication and the inducible promoter of pMB07, but in which we exchanged the wild type *papA5* gene for the mutated version *papA5 F331H*. This PapA5 mutant was identified between several active site mutants of this enzyme and showed to have higher specificity for short-chain alcohols [16]. pMB24 was then transformed into RQ5, yielding RQ5.1 strain. Bioconversion assays of RQ5.1 fed with each BCA exhibited considerable higher yields than RQ5.0 for both BCA-derived MBE: 106% for isobutanol and 52% for isopentanol, respectively (Additional file 2: Fig S1A, B).

### De novo biosynthesis of BCA-derived MBE

**Fig. 1:**
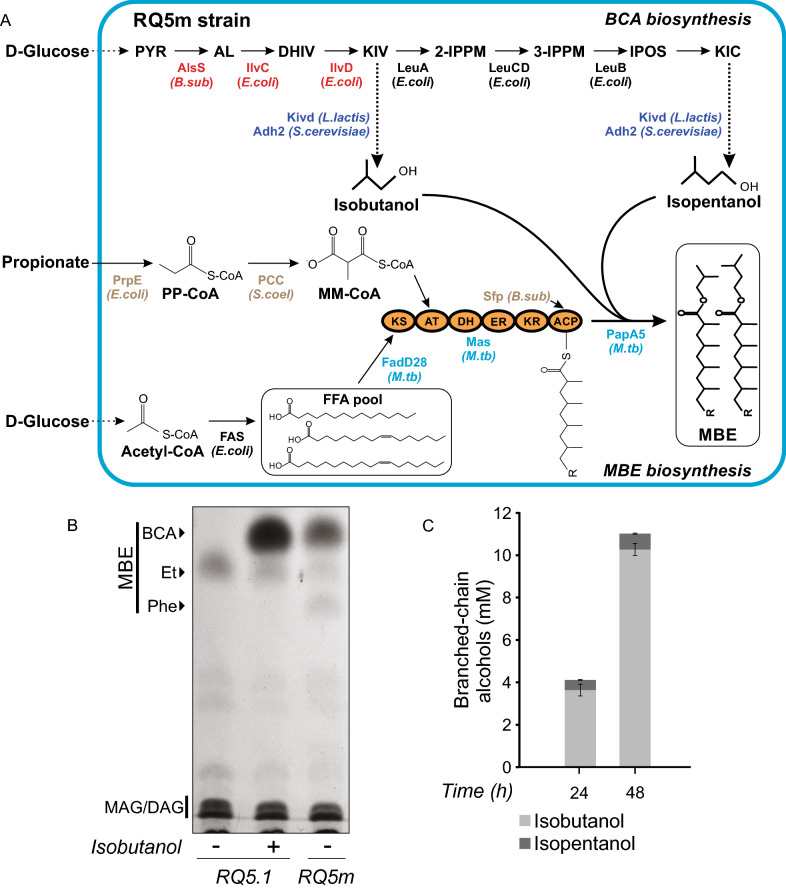
De novo production of branched-chain alcohols derived MBE. **A** Scheme of the branched-chain alcohols (BCA)-derived multi-methyl-branched esters (MBE) biosynthesis pathway in a recombinant *E. coli* strain. Overexpression of the native PrpE (propionyl-CoA ligase) and the heterologous propionyl-CoA carboxylase (PCC) complex (AccA, PccB and PccE subunits) from *S. coelicolor* (*S. coel*) leads to the production of methylmalonyl-CoA (MM-CoA) from exogenous propionate. The MBFA are produced by the MA system from *M. tuberculosis* (*M. tb*) (highlighted in light blue, plasmid pMB24); acyl-AMP ligase (FadD28), mycocerosic acid polyketide synthase (Mas), polyketide-associated protein A5 (PapA5). Sfp is a phosphopantetheinyl transferase of *B. subtilis* (*B. sub*). The BCA isobutanol and isopentanol are produced de novo by the conversion of 2-keto acids into the corresponding alcohols by the expression of *L. lactis* 2-ketoisovalerate decarboxylase Kivd and *S. cerevisiae* alcohol dehydrogenase Adh2 (dark blue, plasmid pIAA12), and the enzymes acetolactate synthase AlsS from *B. subtilis*, and *E. coli* ketol-acid reductoisomerase IlvC and dihydroxy-acid dehydratase IlvD (red, plasmid pIAA11). The genes encoding for the enzymes highlighted in brown are integrated into the chromosome and under the control of T7 promoters. **B** Representative TLC of three independent experiments showing total lipid pattern and MBE production of RQ5.1 and RQ5m (RQ5.1/pIAA11/pIAA12) strains 48 h post induction. (+), isobutanol supplemented to the culture medium at a final concentration of 20 mM. **C** Quantification of BCA production by strain RQ5m at 24 and 48 h post induction. Error bars represent the standard deviation of three independent experiments. PYR, pyruvate; AL, 2-acetolactate; DHIV, 2,3-dihydroxy-isovalerate; KIV, 2-ketoisovalerate; 2-IPPM, 2-isopropylmalate; 3-IPPM, 3-isopropylmalate; IPOS, 2-isopropyl-3-oxosuccinate; KIC, 2-ketoisocaproate; PP-CoA, propionyl-CoA; FAS, fatty acid synthase; FFA, free fatty acids. BCA-MBE, isobutanol and isopentanol-derived MBE; Et-MBE, ethanol derived MBE; Phe-MBE, 2-phenylethanol derived MBE; MAG, monoacylglycerides; DAG, diacylglycerides

In order to de novo produce BCA-derived MBE in *E. coli*, we introduced into the RQ5.1 strain, a pathway that diverts 2-keto acids, intermediates in the biosynthesis of the branched-chain amino acids valine and leucine, into the synthesis of isobutanol and isopentanol. For this, RQ5.1 was transformed with plasmids: (1) pIAA11, that expresses the *B. subtilis alsS* and the *E. coli ilvCD* genes, to increase the production of 2-ketoisovalerate (KIV) [18], a common precursor of both, isobutanol and isopentanol; and (2) plasmid pIAA12 that expresses *kivd* and *ADH2* genes, encoding for the *L. lactis* 2-ketoisovalerate decarboxylase Kivd and the *S. cerevisiae* alcohol dehydrogenase Adh2, respectively, to catalyze the two final enzymatic steps for the BCA biosynthesis pathway [18] (Fig. 1A).

As shown in Fig. 1B, the RQ5.1 strain, that only contains the MA system, is unable to synthesize BCA-derived MBE, unless isobutanol is supplemented to the medium. Instead, the RQ5.1/pIAA11/pIAA12 strain (RQ5m) was capable of producing de novo the expected MBE, although at lower yields than the RQ5.1 strain supplemented with 20 mM isobutanol. The dose response relationship between the external alcohol concentration and the MBE titers (Additional file 2: Fig. S2) suggests that the levels of BCA synthetized de novo by the recombinant RQ5m strain, could be limiting the production of the corresponding MBE. In agreement with this hypothesis, the levels of isobutanol and isopentanol –synthesized through the endogenous LeuACDB pathway of *E. coli* (Fig. 1A)—produced by RQ5m reached a maximum of 11.0 ± 0.3 mM 48 h after the induction of the system (Fig. 1C). Considering the broad substrate specificity of Kivd [17], other alcohols, apart from isobutanol and isopentanol, could have been esterified with the MBFA, for example 2-phenylethanol [16]. Therefore, in order to analyze the alcohol moieties present in the MBE, supernatant samples of the RQ5.1 strain with the external addition of isobutanol and of RQ5m were taken at 48 h post-induction, analyzed by GC–MS and compared qualitatively. As shown in Additional file 2: Fig. S3, differential peaks were detected for isopentanol and 2-phenylethanol (Phe). PapA5 is an enzyme with a relaxed substrate specificity [12, 16] that can use different alcohols as substrate, explaining the detection of Phe-derived MBE and also the ethanol-derived MBE [12] (Fig. 1B).

In this context, we decided to explore a new approach based on the potential benefit of a coculture design. The rationale behind this strategy was to lower the metabolic burden of a unique strain containing the complete MBE biosynthesis system by splitting it into two strains; one strain specialized in producing and releasing the BCA substrates into the medium, and the other one in producing the MBFA and esterifying them with the BCA to synthesize the desired MBE (Additional file 2: Fig. S4). In this way, we expected to obtain an improved BCA production, and consequently, to increase the yields of the corresponding MBE.

### Optimization of the BCA producing strain

One advantage of the coculture system is precisely the possibility to independently optimize each biosynthetic pathway, in this case the production of BCA in one strain and the production of MBFA and of MBE in the other strain.

**Fig. 2:**
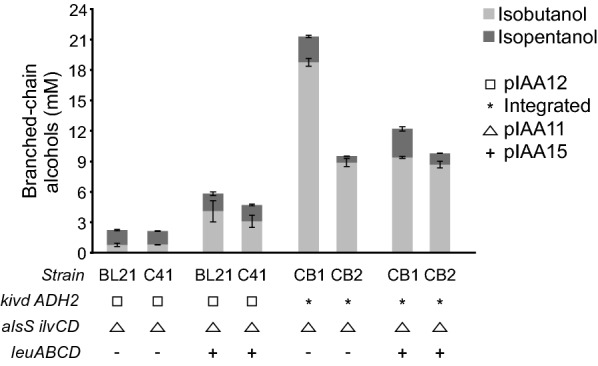
Engineering of the BCA producing strain. Quantification of BCA production by the indicated strains, 48 h after induction. Error bars represent the standard deviation of three independent experiments. (*), the indicated genes are integrated in the chromosome

In order to build an efficient BCA producer strain, we engineered BL21(DE3) and C41(DE3) *E. coli* backgrounds, to divert the 2-keto acids intermediates of the branched-chain amino acid biosynthetic pathway to the corresponding alcohols. To start, both strains were transformed with the high copy number plasmid pIAA12, that expresses *kivd* and *ADH2* genes under the control of the medium-to-high level expression *P*_*LacO-1*_ promoter, and with pIAA11 plasmid, harboring *alsS-ilvCD* genes. As shown in Fig. 2, the resulting BL21/pIAA12/pIAA11 and C41/pIAA12/pIAA11 strains produced similar, although low levels, of isobutanol and isopentanol. To increase production, both strains were further transformed with pIAA15 plasmid to overexpress *leuABCD* genes. The overexpression of these genes was already reported [18] to increase the accumulation of the 2-keto acid KIV, and consequently to enhance isobutanol production. In agreement, a small but noticeable improvement in the total levels of BCA was achieved (Fig. 2), but they were still far away from the ~ 20 mM total BCA needed to reach higher titers of BCA-derived MBE (Additional file 2: Fig. S2).

Therefore, in order to improve the metabolic flux from the 2-keto acids intermediates into the desired alcohols, we hypothesized that the expression levels of Kivd and Adh2 could be a limiting factor, so we decided to overexpress both at higher levels. For this, instead of expressing them from plasmid pIAA12, *kivd* and *ADH2* genes were cloned as a bicistronic operon under the control of the strong *P*_*T7*_ promoter in the pMS7 plasmid and then integrated into the BL21 and C41 *E. coli attB*_*φ80*_ sequence (Additional file 2: Fig. S5.A). The integration of these genes into the chromosome of *E. coli*, should also have a positive effect in the physiology of the cell reducing the metabolic burden caused by the presence of three plasmids corresponding to two different incompatibility groups. The derivative strains were named CB1 and CB2, respectively, and an SDS-PAGE analysis of total proteins extracts confirmed that the expression levels, particularly of Kivd, were higher compared with their expression from plasmid pIAA12 (Additional file 2: Fig. S5.B). Interestingly, a high increase in total BCA levels was reached by both CB1 and CB2 strains carrying plasmid pIAA11 compared with the isogenic BL21 and C41 strains (Fig. 2), confirming our hypothesis. Furthermore, the higher BCA production of CB1/pIAA11 compared with CB2/pIAA11 could also be explained by the higher levels of Kivd and Adh2 in the former strain (Additional file 2: Fig. S5.B, C). Growth curves of the different strains constructed show that the maintenance of pIAA12 and pIAA11 plasmids did not cause an effect in the growth profile (Additional file 2: Fig. S6). Altogether, these results would suggest that the BCA biosynthetic pathway is highly dependent on the expression levels of these enzymes, and that this is a critical parameter to be fined tuned to reach higher BCA production.

Finally, the overexpression of *leuABCD* in both CB1/pIAA11 and CB2/pIAA11 did not produce a significant improvement in total BCA titers, as happened in BL21 and C41 strains (Fig. 2). In fact, even though there was a small increase in isopentanol titers, it had a negative impact on isobutanol production, particularly strong for CB1. We hypothesized that because of the higher levels of Kivd and Adh2, that can drain not only the pool of the intermediate metabolite KIV to isobutanol but also the 2-ketoisocaproate (KIC) pool (precursor of leucine) to isopentanol, lower levels of free leucine are expected in the cell. In consequence, *leuA* gene product could be partially relieved from its feedback inhibition by leucine [18]. Therefore, the increased LeuA activity plus its overexpression with plasmid pIAA15, could then force the metabolic flux from KIV to KIC, explaining the increase in isopentanol and the decrease in isobutanol.

Based on these results, CB1/pIAA11 strain (from now on named CB1.1) reached a total of 21.3 ± 0.3 mM BCA, the highest titers of BCA among the engineered strains (Fig. 2), even higher than the 20 mM isobutanol externally added to the culture media for RQ5.1 monoculture; so it was chosen as the best partner to establish a coculture-based system.

### General strategy and initial coculture conditions

**Fig. 3:**
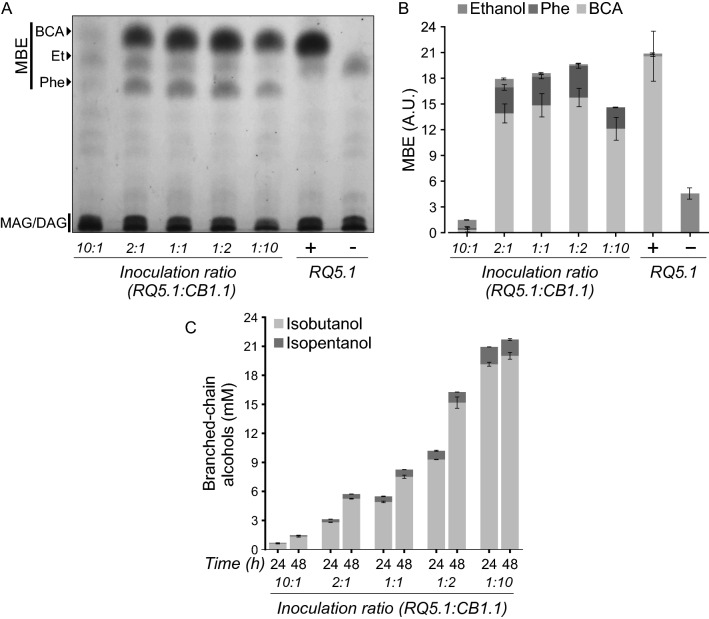
MBE and BCA production by the RQ5.1/CB1.1 coculture system. **A** Representative TLC of three independent experiments showing MBE production by the RQ5.1/CB1.1 coculture system 48 h post induction, varying the inoculation ratio between both strains. Isobutanol was supplemented to the culture medium at a final concentration of 20 mM for bioconversion assays of the RQ5.1 strain (positive control). BCA-MBE, isobutanol and isopentanol-derived MBE; MBE-Et, ethanol derived MBE; MBE-Phe, 2-phenylethanol derived MBE; MAG, monoacylglycerides; DAG, diacylglycerides. **B** Densitometric analysis of the MBE spots of the TLC. **C** Quantification of BCA production for each of the cocultures at the indicated time points. Error bars represent the standard deviation of three independent experiments

Based on the previous analyses, the coculture system was then constituted by CB1.1 strain as the BCA producer partner and RQ5.1 as the MBFA/MBE producer strain. The initial optimization of the system started by varying the inoculation ratio of RQ5.1 and CB1.1 cells in the bioconversion assays from 10:1, 2:1, 1:1, 1:2, 1:10; respectively. The set-up of the initial parameters for the cocultures, such as induction time and inducer concentration, were selected based in monoculture bioconversion experiments (2 h after inoculation –OD_600_ ~ 0.5– and 0.1 mM IPTG). Cocultures were incubated after induction for 48 h at 23 °C and total lipids were extracted and analysed by TLC. As shown in Fig. 3A, the coculture strategy was successful in producing BCA-derived MBE without the external addition of the alcohol. Densitometric quantification of MBE (see “Methods” section) (Fig. 3B) indicated that the highest production was achieved at the initial inoculation ratio of 1:2. Further, the initial inoculation ratios of 10:1 and 1:10 were detrimental for MBE titers (Fig. 3B), most probably due to the low alcohol production levels in the first mix (Fig. 3C) and to the underrepresentation of the MBE producer strain RQ5.1 in the second one. These results indicated, as expected, that the inoculation ratio is a highly sensitive parameter, and that a proper alcohol titer is needed to achieve high MBE production levels. In fact, when the BCA concentration in the supernatants of each of the cocultures was assayed at 24 and 48 h, we found that the BCA levels were directly proportional to the higher representation of the CB1.1 strain in the inoculation mix (Fig. 3C). Therefore, this result indicated that the lowest levels of MBE in the 1:10 ratio occurred due to the limitation in the number of bacteria capable of synthesizing the esters.

It is worth mentioning that Phe-derived MBE was also synthetized by the coculture (Fig. 3A). Furthermore, 2-phenylethanol was found in the supernatant of the coculture samples, and although it was not quantified, the overexpression of Kivd and Adh2 in CB1.1 strain appears to have conducted to an enhanced production of this alcohol too, as can be seen by comparing the ratio between the different MBE produced (Figs. 3A, B and Fig. 1B).

### Optimization of the coculture BCA-derived MBE productivity by response surface methodology

As shown in the previous section, the inoculation ratio between RQ5.1/CB1.1 strains was a variable that significantly influenced MBE titers of the coculture in shake flasks. Another important bioconversion parameter to consider was the induction of the complete MBE production system; specifically, the time of addition and the concentration of the inducer (IPTG). The optimization of these parameters has been shown to have a strong impact in the titers of the final products [21].

To determine the combination of these three variables –i.e. inoculation ratio, induction time and inducer concentration—that maximized the BCA-derived MBE production by the coculture, the optimization of one variable at a time (OVAT) would have led to suboptimal solutions, since it does not take into account the interactions among the different variables [22]. In a coculture, we expected the existence of significant interactions between these variables. For example, even slight differences in the growth rate between the constituent strains of the coculture, could lead to the existence of strong interactions between the inoculation ratio and the induction time of the system. This is, a coculture with an inoculation ratio that favors the slow growing strain might need more time to reach the best induction point, and the other way around.

Therefore, we decided to carry out a systematic optimization procedure using response surface methodology (RSM) which has several advantages [23]. First, it is based on statistical experimental designs that change several variables simultaneously, so the interactions between them are considered. Second, these designs aim to maximize the information gained by conducting as few experiments as possible. In this case, the central composite design (CCD) chosen allowed us to evaluate each variable at five levels with only 34 experiments (counting replicates), a very small number compared, for example, with a full factorial design that would have required 5^3^ = 125 experiments (without counting replicates) to explore all possible combinations of the variables at five levels each. Finally, the results of those few experiments would allow us to sample, and then empirical model, the global production landscape of the coculture for the three independent variables under study.

Additional file 1: Table S1 shows the production of the BCA-derived MBE normalized by OD_600_ (the response to be optimized) obtained while varying the cocultures under the conditions established by the CCD. This response was fitted to a polynomial model, using forward selection regression to estimate the best one, and the following reduced cubic model was obtained:

$${Y}_{1}= 23.91-2.5 A-1.32B+1.19C-2.33 AB-0.078 AC+0.68 BC-5.62 {A}^{2}-4.42 {B}^{2}-5.48 {C}^{2}-3.3 ABC-1.6 {A}^{2}B+ 1.45 {C}^{3}$$

where *Y*_*1*_ represents the BCA-derived MBE production (A.U., arbitrary units determined by densitometric measures of MBE in TLC analysis); *A*, *B* and *C* are the coded values of the independent variables: inoculation ratio (% of RQ5.1), inoculation time and inducer concentration, respectively.

The adequacy of the model was checked by analysis of variance (ANOVA) (Additional file 1: Table S2). A *p*-value of the model < 0.05 shows that it was significant, and a determination coefficient (R^2^) of 0.958 and a non-significant Lack of Fit (p = 0.38), shows that the model fitted well the experimental data. Also, an ${\mathrm{R}}_{\mathrm{prediction}}^{2}$ of 0.867 indicates that the overall predictive capability of the model was very satisfactory [23]. The ANOVA also indicated that even though the only linear significant term was the inoculation ratio (*A*), all the quadratic terms (*A*^*2*^, *B*^*2*^, and *C*^*2*^) were significant, especially the double interaction inoculation ratio-induction time (*AB*) and the triple interaction *ABC*. The significance of those interactive terms confirms the presence, in the coculture, of strong interactions between the three variables and highlights the importance of performing the optimization with the RSM multivariate approach.

**Fig. 4:**
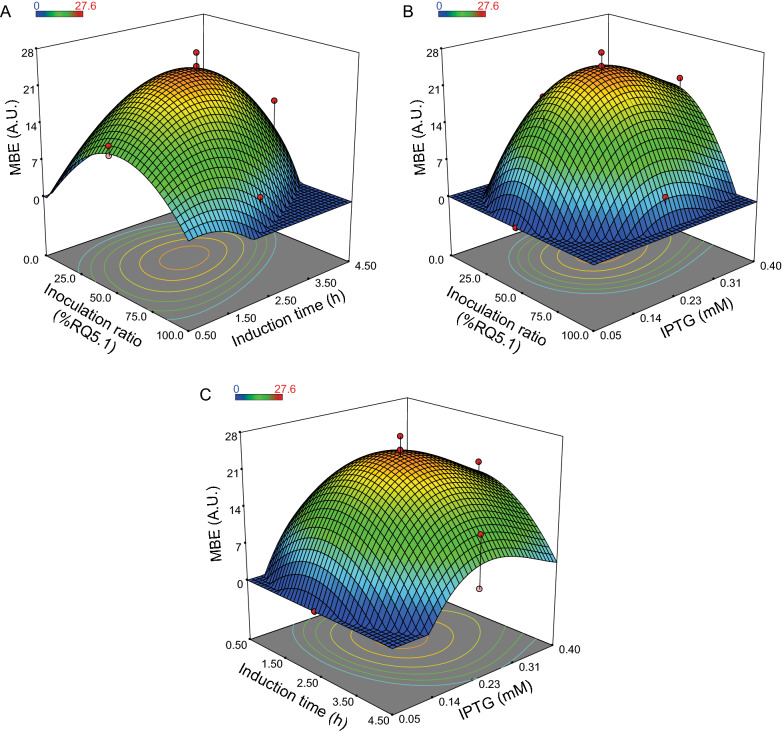
Optimization of the BCA-derived MBE production by the coculture using RSM. **A**–**C** Response surface plots for BCA-derived MBE production as a function of the three variables under study: inoculation ratio (%RQ5.1), induction time (h), and inducer (IPTG) concentration (mM). These plots were obtained for a given pair of variables, while maintaining the other one fixed at its zero value. Contour plots are projected on the *x*–*y* plane. The red and pink points correspond to experimental design points. Interactive versions of the response surface plots at: https://github.com/ferbracalente/E.-coli-coculture

With this model, the response surface for the BCA-derived MBE production, as a function of the three independent variables was built, and the production landscape of the coculture could easily be explored (Fig. 4). As shown in Fig. 4(A-C), when the variables are closed to their center values (zero levels, Additional file 1: Table S3), the response maximizes, and it decreases as the variables move to the edges. When numeric optimization was performed, the predicted value corresponding to a maximum of BCA-derived MBE production was 24.3 A.U. (densitometric measurement of MBE expressed in arbitrary units) and the optimum experimental conditions were: 45% RQ5.1 at inoculation, induction time of 2.4 h and IPTG concentration of 0.24 mM. In concordance with the response surface plots, this calculated optimum point is almost the center point of the design (50% RQ5.1, induction time of 2.5 h, and 0.23 mM IPTG). Therefore, we considered the center point of the design to be the optimum of the system and the arithmetic mean of the response for the six experimental replicates of this center point (Additional file 1: Table S1) was computed. Thus, we obtained an experimental maximum of 23.7 ± 2.3 A.U., showing an excellent agreement with the theoretical maximum predicted by the model.

Since the productivity of the system also depends on the final OD_600_ reached by each culture, we analyzed that the central region, where the MBE production per cell was predicted to be maximum also had appropriate values of cell density. The OD_600_ response (Additional file 1: Table S1) was then fitted to a reduced cubic model using backward elimination regression (Additional file 1: Eq. S1), with good statistical indicators (i.e. R^2^ = 0.91 and a non-significant Lack of Fit) when it was checked by ANOVA (Additional file 1: Table S4), and the response surface plots as a function of the three variables under study were built (Additional file 2: Fig. S7 A–C). Finally, this model for the final OD_600_, and the previous model for the BCA-derived MBE production of the coculture were used to perform a multiresponse optimization using the *desirability* function (see “Methods” section), with the goal to maximize both responses simultaneously. Each response was transformed to an individual desirability function *d*_*i*_, then combined in a global desirability function *D* and its response surface was obtained (Additional file 2: Fig. S7 D–F). The experimental conditions to a maximum in the desirability function (*D* = 0.79) were: 53.9% RQ5.1 at inoculation, induction time of 2.51 h, and IPTG concentration of 0.21 mM, with a predicted OD_600_ = 8.48 and a predicted BCA-derived MBE production = 22.95 A.U. This result and the response surface for *D*, confirmed that to reach the coculture’s optimum, in terms of both MBE production and final cell density, the variables needed to be close to their zero levels and that suboptimal behaviors are observed when the system moved away from that combination.

After optimization by RSM, the coculture reached a production of 20.1 ± 1.5 mg/L of BCA-derived MBE. This value represented a 12.5% increase when compared with the best production yield obtained in the previous section, 17.9 ± 0.5 mg/L, for an inoculation ratio of 1:2 (33% RQ5.1), induction time of 2 h and 0.1 mM IPTG (Additional file 2: Fig. S8). Furthermore, this maximum titer reached after optimization was 45% higher than the one obtained for the monoculture strategy, and only a 7% lower than the one obtained for RQ5.1 with the external addition of 20 mM isobutanol (21.7 ± 0.2 mg/L) (Additional file 2: Fig. S8). These results indicated that the coculture system fully achieved our objective of designing an efficient bioprocess to de novo produce BCA-derived MBE from only glucose and propionate.

### Scale up: high cell density fed-batch fermentation of the coculture for MBE production

**Fig. 5:**
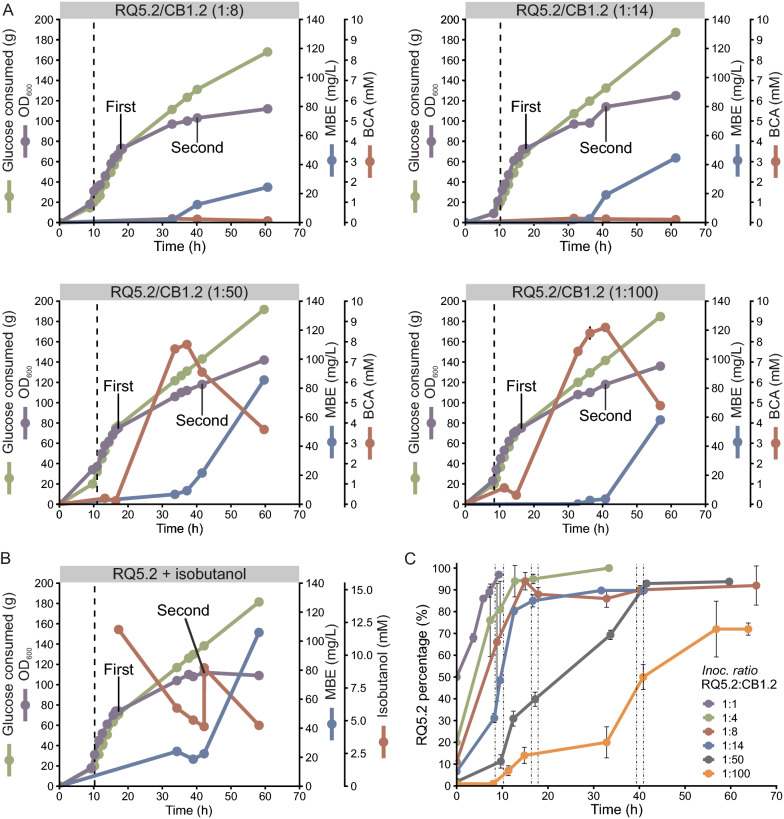
Scale-up of the MBE production using the *E. coli* coculture in high-cell density fed-batch fermentations. **A** and **B** Time profiles of MBE and the intermediate BCA –isobutanol and isopentanol– concentrations, cell density (OD_600_) and glucose consumed are represented for the coculture RQ5.2/CB1.2 at different inoculation ratios (indicated in the grey box on top of each plot) (**A**) and for the RQ5.2 strain alone with exogenous addition of isobutanol (**B**). The vertical dashed lines indicate the end of the batch phase and the start of the fed-batch phase. “First” and “Second” labels indicate the time when IPTG, propionate and isobutanol (only in **B**) were added to the culture. OD_600_ and glucose consumed are depicted in the same axis. **C** Time profile of RQ5.2 percentage in the fed-batch bioreactor cocultivation at different inoculation ratios. The two dashed-dotted lines on the left delimit the time frame when the batch phase was concluded, the two on the middle the time frame when the first induction was carried out, and the two on the right the time frame of the second induction. Error bars represent the standard deviation of three technical replicates

Once we achieved a satisfactory MBE production using the engineered coculture system in small *shake flask* fermentations, we took a step forward towards the scalability of the system. For this, high cell density fed-batch fermentations of the coculture were carried out in a 2 L bioreactor following a procedure similar to the one developed by Menendez-Bravo et al*.* for a single MBE-producing strain and with octanol as the externally supplied alcohol [13]. The main difference of our system, compared with that developed by Menendez-Bravo et al*.,* was the presence of the two constituent strains of the coculture, RQ5.2 and CB1.2 (Table 1, derivative strains constructed for cell counting and plasmid resistance compatibility in cocultures, see “Methods” section). Briefly, the two strains were inoculated in 1 L of M9 medium and 20 g/L glucose for an initial batch phase of growth until all the glucose was consumed. Then, glucose was fed to the bioreactor and at OD_600 _ ~ 75, the MBE producing system was induced with the addition of a pulse of IPTG and propionate. 24 h later, a second pulse of IPTG and propionate was carried out, and the fed-batch phase was continued until the process was concluded at  ~ 60 h of total duration. Throughout the fermentation process, we determined the following parameters: growth at OD_600_, consumption of glucose, BCA and BCA-derived MBE concentration (Fig. 5A, B), and finally the strain-to-strain ratio in order to follow the dynamic of the population (Fig. 5C).

**Table 1:** Plasmids and strains used in this study

Plasmid	Description	Source
pET28a	Vector for protein expression with N-terminal His tag under P_T7_; Km^R^	Novagen
pCA30	Expression vector with p15A replication origin and P_T7_; Cm^R^	[13]
pAH123	Helper plasmid for expression of phage φ80 integrase*;* Ap^R^	[26]
pAH162	Plasmid containing the *attP* sequence of phage φ80; Tc^R^	[26]
pBAD33	Vector for protein expression under P_ARA_; Cm^R^	[29]
pJR22	pET28a-derived vector containing gene *papA5 F331H*; Km^R^	[16]
pSC100	pET28a-derived vector containing genes *fadD28* and *mas*; Km^R^	Santiago Comba, personal communication
pMB07	pET28a-derived vector containing genes *fadD28*, *mas* and *papA5*; Km^R^	[13]
pMB23	pET28a-derived vector containing genes *fadD28*, *mas* and *papA5 F331H*; Km^R^	This work
pMB24	pMB23 derived plasmid; Cm^R^	This work
pIAA11	Expression vector harboring *E. coli ilvCD* genes and *Bacillus subtilis alsS* gene under P_LlacO_^−1^; Km^R^	[18]
pIAA12	Expression vector harboring *Lactococus lactis kivd* gene and *Saccharomyces cerevisiae ADH2* under P_LlacO_^−1^; Ap^R^	[18]
pIAA15	Expression vector harboring *E. coli leuABCD* genes under P_LlacO_^−1^; Cm^R^	[18]
pMS7	pAH162-derived vector containing the bicistronic operon *kivd-ADH2*, under P_T7_; Tc^R^	This work
pGFP	pBBR1 ori; expressing the green fluorescent protein (GFP) from *Aequorea victoria* under P_Lac_; Cm^R^	Gonzalo Tulin, personal communication

Strain	Description	Source
DH5α	*E. coli* K12 F^*−*^* lacU169 (Φ80lacZΔM15) endA1 recA1 hsdR17 deoR supE44 thi*^*−*^*1 l2 gyrA96 relA1*	[30]
BW25142	*lacI*^q^ *rrnB3 ΔlacZ4787 hsdR514* DE*(araBAD)567* DE*(rhaBAD)568 ΔphoBR580 rph-1 galU95 ΔendA9 uidA(ΔMlu*I)*::pir-116 recA1*	[26]
RQ5	*E. coli* F*- omp*T *gal dcm lon hsd*S_B_ (r_B_^−^, m_B_^−^) λ(DE3) *prpRBCD:: P*_*T7*_*-sfp-P*_*T7*_*-prpE ygfGH::P*_*T7*_-*accA2*-*pccE*-*pccB*-*T7term ΔfadE*	[13]
RQ5.0	RQ5 harboring pMB07	This work
RQ5.1	RQ5 harboring pMB24	This work
RQ5m	RQ5.1 harboring pIAA11 and pIAA12	This work
RQ5.2	RQ5 harboring pMB23 and pGFP	This work
BL21(DE3)	*E. coli* F*- omp*T *gal dcm lon hsd*S_B_ (r_B_^−^, m_B_^−^) λ(DE3)	Novagen
C41(DE3)	*E. coli* F*- ompT gal dcm lon hsd*S_B_ (r_B_^−^, m_B_^−^) λ(DE3)	[31]
BL21/ pIAA12/ pIAA11	BL21(DE3) harboring pIAA12 and pIAA11	This work
C41/ pIAA12/ pIAA11	C41(DE3) harboring pIAA12 and pIAA11	This work
BL21/ pIAA12/ pIAA11/ pIAA15	BL21(DE3) harboring pIAA12, pIAA11 and pIAA15	This work
C41/ pIAA12/pIAA11/pIAA15	C41(DE3) harboring pIAA12, pIAA11 and pIAA15	This work
CB1	BL21(DE3) *attB*_*φ80*_::pMS7	This work
CB2	C41(DE3) *attB*_*φ80*_::pMS7	This work
CB1.1	CB1 harboring pIAA11	This work
CB2/pIAA11	CB2 harboring pIAA11	This work
CB1/pIAA11/pIA15	CB1 harboring pIAA11 and pIAA15	This work
CB2/ pIAA11/pIAA15	CB2 harboring pIAA11 and pIAA15	This work
CB1.2	CB1.1 harboring pBAD33	This work

*Ap*^*R*^ ampicillin resistance, *Km*^*R*^ kanamycine resistance, *Cm*^*R*^ chloramphenicol resistance, *Tc*^*R*^ tetracycline resistance

To initiate the scale up process, we inoculated the bioreactor with the strains RQ5.2 (RQ5 harboring pMB23 and pGFP) and CB1.2 at a 1:1 ratio, by diluting each strain to an initial OD_600_ of 0.001 in the bioreactor. Surprisingly, neither MBE nor the intermediate BCA were detected after 60 h, which led us to hypothesize that certain instability of the population might have occurred. In fact, RQ5.2 rapidly took over the culture reaching 97% of the whole population at the end of the batch phase (Fig. 5.C). Even though this uneven growth rate was unexpected for the two *E. coli* strains, to confirm this apparently higher growth rate of RQ5.2 in glucose compared with CB1.2, we performed growth curves of each strain in small-scale batch cultures at two different initial OD_600_ (0.02 and 0.001) in microplates containing M9 medium with 20 g/L glucose and in flasks containing 10 mL of the same medium (Additional file 2: Fig. S9.A, B). Under these conditions, CB1.2 showed a slightly longer lag phase and slower growth rate compared with RQ5.2, however the differences on these two variables were minor. When these variables were independently evaluated for each strain in a bioreactor (initial OD_600_ of 0.001 in 1 L M9 medium and 20 g/L glucose) the differences in the growth profile of both strains were markedly distinct (Additional file 2: Fig. S9.C). While RQ5.2 started the exponential phase of growth immediately after inoculation with an estimated specific growth rate µ = 0.71 h^−1^ and consuming all the glucose in less than 10 h, CB1.2 exhibited a very prolonged lag phase of  ~ 15 h, followed by an exponential phase with an estimated µ = 0.25 h^−1^. As the dilution to an initial OD_600_ of 0.001 for the CB1.2 strain in small-scale in microplate or in flask did not show an extended lag phase like it was observed in bioreactors, we hypothesize that this unexpected behavior could be associated with the impact that the physical parameters found in the fermenter might have in the expression of the heterologous genes in this strain, leading to a premature unbalance in the amino acid metabolism. Further investigations will be needed in the future to understand this phenomenon.

To compensate this difference in growth rate, we changed the proportions of the two strains in the initial population in the bioreactor, moving to inoculation ratios that favored the slow growth rate of the CB1.2 strain. Fixing RQ5.2 at an initial OD_600_ of 0.001, we first tried inoculating 4 times more of CB1.2, but again neither MBE nor BCA were detected after 60 h of growth. Although this change allowed the alcohol producing strain to be more represented at the end of the batch phase, RQ5.2 continued its fast growth during the fed-batch phase, dominating the whole population at a 95% at the time the first induction was performed (Fig. 5C), explaining the absence of BCA, and therefore of MBE, production. We then performed experiments with 1:8 and 1:14 (RQ5.2/CB1.2) inoculation ratios. In both cases, the bioconversion was successful. Even though the total BCA detected in the supernatant of the cultures were very low, 0.18 mM for 1:8 ratio and 0.21 mM for 1:14 ratio; MBE derived of these alcohols were produced by the cells with a final titer of 24 mg/L and 44 mg/L, respectively (Fig. 5A, first and second plots), indicating that the alcohol concentrations reached at these ratios were limiting the production of the final product. As expected, the more CB1.2 at the start of the process, the less percentage of RQ5.2 at the end of the batch phase (Fig. 5C). More importantly, CB1.2 represented a 10% for 1:8 and 15% for 1:14 of the whole population at the time of the first induction, and stabilized around the same percentage for the rest of the process (Fig. 5C); explaining the limited success of these fermentations in terms of MBE productivity.

Based on these results, we evaluated two more inoculation ratios increasing the proportion of CB1.2 in the initial population. At a 1:50 (RQ5.2/CB1.2) ratio, the final titer of BCA-derived MBE was 86 mg/L, a twofold improvement over the system inoculated at 1:14. The concentration of total BCA during the fermentation, that appeared to be limiting the production of MBE in previous experiments, was also higher, reaching a peak of 7.9 mM at 37 h, after which it started to decrease, most probably due to a dilution effect caused by the fed-batch process, and also to the consumption of these substrates by the RQ5.2 strain, as indicated by the sustained increase in MBE production (Fig. 5A, third plot). As shown in Fig. 5C, RQ5.2 represented only a 10% of the total cells by the end of the batch phase, while at the time of the first induction it already represented  ~ 40% of the coculture, and by the second induction it had gained the population reaching and stabilizing at  ~ 90% after 41 h until the end of the process. When the fermentation process was started with an inoculation ratio of 1:100 (RQ5.2/CB1.2), a maximum of total BCA concentration of 8.7 mM was obtained, however this was not reflected in the MBE production, that only reached 58 mg/L (Fig. 5A, forth plot). This result is explained by the underrepresentation of the MBE producer strain during almost the entire process. In fact, RQ5.2 reached only  ~ 14% of the total population at the first induction time and a 50% at the second induction after 40 h of growth, to finally stabilize at a 75% very close to the end of the process (Fig. 5C). It is clear that only the fermentation with an inoculation ratio of 1:50 reached the induction time with a well-balanced population, with a 40%-60% of each strain, and it was reflected in the productivity of the system: acceptable levels of the BCA were synthetized, and the highest MBE titer was achieved. Thus, we have identified, in fed-batch processes, the percentage composition of the population at the induction time, as a key factor for the successful production of the final product. Since the optimum composition at induction was achieved by changing the inoculation ratio and monitoring the dynamic of the population over time, this demonstrates that the inoculation ratio is still a very powerful tool to tune up the coculture, not only at small scale in batch cultures, but also in long scaled-up fed-batch processes. Regarding the dynamic of the two strains in the coculture throughout the fermentation process, we noticed that the higher the RQ5.2/CB1.2 inoculation ratio, the faster RQ5.2 gain the population of the culture; this result was consistent with the higher growth rate of this strain compared with that of CB1.2 (Additional file 2: Fig. S9). However, at lower ratios, the growth rate of RQ5.2 was clearly affected by the overrepresentation of the BCA producer strain, suggesting a strong competition of the strains for the substrates.

Finally, to evaluate how close the coculture in the bioreactor was to its best performance, we carried out, as a control, a fed-batch fermentation with only the RQ5.2 strain, with externally supplied isobutanol. For this, two pulses of this alcohol were added, one of 12 mM when the first induction was performed, and another one of 5 mM at the time of the second induction. The final isobutanol derived MBE titer obtained was 106 mg/L (Fig. 5B), a 17% more than the 86 mg/L produced by the coculture with an inoculation ratio of 1:50. This indicated that the coculture strategy was successfully implemented to produce BCA-derived MBE, from only glucose and propionate and dispensing the external addition of the alcohols. Several microbial platforms have been developed to produce a variety of lipid-derived compounds with very different yields. For example, for FAEE the production ranges from titers as low as 6.3 mg/L in *Saccharomyces cerevisiae* [24] to 92.2 mg/L of C14 FAEE in *E. coli* [25] and up to 560 mg/L of C12-C14 FAMEs by overexpression of the *Drosophila melanogaster* methyltransferase DmJHAMT in *E. coli* [6]. Therefore, the levels of MBE produced by our platform fit within the average titers that have been reached for other less complex esters.

## Conclusions

An *E. coli* strain was highly engineered to synthesize de novo BCA and MBFA, and to use these biosynthetic building blocks for the production of non-natural esters with multiple methyl branches. The low productivity of these molecules by a monoculture containing both biosynthetic pathways was overcome by the design of a coculture system, which allowed to separate the BCA and MBFA pathway into two different strains, and optimize them independently. The inoculation ratio between both strains, and other fermentation parameters of the coculture system were successfully optimized by RSM. We proved this multivariate statistical methodology to be effective to sample and empirical model the production landscape of a coculture with a very limited number of experiments, saving time and cost, and providing valid information to improve the productivity of the system. Finally, the coculture system was scaled-up to produced BCA-derived MBE in high-cell density fed-batch fermentations by fine-tuning the inoculation ratio between the constituent strains, showing that optimizing this parameter is essential at any scale.

## Methods

### Plasmids construction

All the oligonucleotide primers used in this work are listed in Table S5, Additional file 1, and all plasmids in Table 1. *E. coli* DH5α was used for routine cloning and subcloning.

For the construction of plasmid pMB23, that expresses *M. tuberculosis papA5 F331H* (mutant version of *papA5*), *fadD28* and *mas* genes as a tricistronic operon under the control of T7 promoter (*P*_*T7*_), *papA5 F331H* gene was isolated as a *Xba*I/*Spe*I digestion fragment from plasmid pJR22 [16]. Then, it was cloned into the *Xba*I site of the plasmid pSC100 (Santiago Comba, personal communication), a pET28a-derived vector containing *fadD28* and *mas* genes. Correct orientation of the insert was checked with restriction enzymes.

For the construction of plasmid pMB24, the Cm^R^ cassette sequence from pBAD33 vector was amplified by PCR using the pBAD_Cm F/R primer pair. After DNA purification, it was digested with *Nsi*I and ligated into the *Nsi*I sites of plasmid pMB23, interrupting the Km^R^ cassette sequence and yielding pMB24.

For the construction of the plasmid pMS7, *L. lactis kivd* and *S. cerevisiae ADH2* coding sequences were amplified by PCR using plasmid pIAA12 as template and kivD_Fw/Rv and ADH2_Fw/Rv primer pairs, respectively. The DNA fragments were cloned into pJET1.2 vector (Thermo Fischer) and checked by DNA sequencing (Maine University, DNA sequencing facility, USA). The resulting plasmids were digested with *Nde*I*/Spe*I and the restriction fragment cloned into *Nde*I*/Spe*I restriction sites of plasmid pET28a (to express both genes as 6xHis-tag fusion proteins), yielding plasmids pMS3 for *kivd* and pMS4 for *ADH2*. Then, pMS4 was digested with *Xba*I*/Hind*III, and the restriction fragment containing *ADH2* gene cloned downstream of *kivd* gene, in the *Spe*I*/Hind*III sites of pMS3. After that, in order to have the resulting operon *kivd-ADH2* under the control of *P*_*T7*_, it was isolated as a *Xba*I*/Spe*I digestion fragment and cloned into pCA30 *Spe*I site. Correct orientation of the insert was checked with restriction enzymes. Finally, the *P*_*T7*_*-kivd-ADH2* operon was isolated with *Xba*I*/Spe*I and ligated to the integrative pAH162 plasmid digested with *Spe*I, yielding plasmid pMS7. pMS7 was maintained in a *pir-116* permissive host, *E. coli* BW25142.

### Strains construction

All the strains used in this work are listed in Table 1. RQ5 strain was transformed by electroporation with plasmid pMB07 to give rise to RQ5.0 strain, with plasmid pMB24 to yield RQ5.1 strain, and with plasmids pMB23 and pGFP to yield RQ5.2 strain. CB1 and CB2 strains were obtained by integration of pMS7 plasmid into the *attB*_*φ80*_ site of *E. coli* BL21(DE3) and C41(DE3) genomes, respectively, as described in [26]. A scheme of the strategy can be found in Fig. S5.A, Additional file 2. Single copy integration was checked by PCR using P1, P2, P3 and P4 primers [26]. Correct protein expression was checked by Western blot, using monoclonal anti-6xHis antibodies (QIAGEN) and anti-mouse IgG-horseradish peroxidase (HRP) conjugated as secondary antibodies, and visualized by chemiluminescence (Additional file 2: Fig S5.C).

### Growth media

*E. coli* strains were grown either on solid or in liquid Luria–Bertani medium (LB; 10 g Bacto Tryptone, 5 g yeast extract, and 10 g NaCl per liter) at 37 °C for cloning routines, plasmid propagation and colony isolation. For all bioconversion assays strains were grown in a modified M9 medium (M9; 6 g Na_2_HPO_4_, 3 g KH_2_PO_4_, 1 g NH_4_Cl, 0.5 g NaCl, 2 mM MgSO_4_, 0.1 mM CaCl_2_ per liter of water) containing 10 g/L of glucose and 2 g/L of yeast extract. The following antibiotics were added when needed: 150 µg/mL ampicillin (Ap), 50 µg/mL kanamycin (Km), and 20 µg/mL chloramphenicol (Cm).

### Small-scale bioconversion assays

For all experiments, a single colony from a fresh LB-plate of the indicated strain was inoculated into 2 mL of M9 medium with the appropriated antibiotics and grown overnight (ON) in an orbital shaker at 37 °C and 200 rpm.

For MBE production in monoculture experiments, the ON culture of the appropriate RQ5 derived strain was diluted 1/50 into 10 mL of fresh M9 medium and grown at 37 °C and 200 rpm. At the mid-exponential phase, cultures were induced with 0.1 mM isopropyl-β-d-thiogalactopyranoside (IPTG) and supplemented with 20 mM sodium propionate. Only when indicated, isobutanol was also added at a final concentration of 20 mM.

For coculture experiments, an ON culture was grown for each of the constituent strains, separately. Next day, optical density of the ON cultures was measured at 600 nm (OD_600_) in 96-well plates using a microplate reader (BioTek Epoch 2), and then mixed to inoculate 10 mL of fresh M9 medium, diluting the indicated MBE strain to a fixed initial OD_600_ = 0.02 and varying the indicated BCA strain according to the inoculation ratio used in each experiment. Cultures were grown in an orbital shaker at 37 °C and 200 rpm, until they were induced at the indicated time and with the indicated concentration of the inducer (IPTG) and supplemented with 20 mM sodium propionate.

For both monocultures and cocultures, cultures were further incubated after induction for another 48 h at 23 °C and 200 rpm. Supernatant samples were taken to analyze BCA production at 24 and 48 h. At 24 or 48 h, to analyze MBE production, the final OD_600_ of the culture was measured, cells corresponding to an equivalent of 1 mL OD_600_ = 10 were harvested by centrifugation, and total lipids were extracted from cell material according to the Bligh and Dyer method [27] and analyzed by TLC.

### TLC analysis

Total lipid extracts were analyzed by TLC on silica gel 60 F254 plates (± 0.2 mm, Merck) using the solvent system hexane/diethyl-ether/acetic acid (90: 3:75: 1, v/v/v) for the mobile phase. Lipids were visualized by dipping the plate for 1 s in a solution 5% CuSO_4_ in 8% aqueous H_3_PO_4_, drying it for 10 min at room temperature and then placing it into a 120 °C oven for 30 min. A calibration curve ranging from 10 to 40 µg of cetyl-palmitate (CP) was used as external standard. Then, a digitalized image of the stained TLC was taken, and densitometric quantification of the spots corresponding to MBE and CP was performed using ImageJ 1.52a. The amount of MBE produced by the different strains in monocultures or cocultures systems was expressed as arbitrary units (A.U). Using the equation obtained by linear regression from the CP calibration curve, the amount of MBE obtained from processing equivalent amount of cells (1 mL OD_600_ = 10) was expressed in mg/L.

### BCA analysis and quantification using GC–MS

BCA were extracted from 500 µL of supernatant samples (representing the unevaporated fraction of the alcohols) of the cultures with 1 mL dichloromethane. Then, GC–MS analyses were performed using a SUPELCOWAX-10 column (28 m long × 0.25 mm internal diameter × 0.25 µm thickness, Supelco) on a Shimadzu GCMS-QP2010 Plus (Shimadzu Co., Kyoto, Japan), operating at an ionization voltage of 70 eV with a range of 30 to 600 Da. The column temperature was programmed as follows: 35 °C for 5 min, increasing 25 °C/min to reach 120 °C, then increasing 60 °C/min to reach 230 °C and finally held for 1 min. Helium was used as carrier gas at a linear velocity of 37.5 cm/s. The injection volume was 1 μL and a division ratio of sample, or split, of 1/10. The injection temperature was maintained at 150 °C and the ion source at 230 °C. Isobutanol and isopentanol were identified via GC retention times and mass spectra, and quantified by normalizing to an internal standard (butanol) and comparing against a standard curve for each alcohol.

### Experimental design, statistical analysis and multiresponse optimization

To optimize the BCA-derived MBE production of the coculture in shake flasks, a central composite design (CCD) was used to determine the design points for applying the response surface methodology (RSM). The three independent variables (factors) studied were: A: inoculation ratio, expressed as a percentage of the downstream strain RQ5.1 (% RQ5.1); B: induction time, and C: inducer (IPTG) concentration. These variables were evaluated at five levels each (Additional file 1: Table S3). A total of 34 experiments were needed to fulfill six replicates of the central point and two replicates for each factorial and star points. In addition, the 34 experiments were carried out in two different blocks (day 1 and 2) to avoid work overload (Additional file 1: Table S1). All experiments were performed in random order to minimize the effects of uncontrolled factors that may introduce a bias on the measurements.

To evaluate the productivity of the system, two response variables were measured for each experiment: (1) BCA-derived MBE production normalized by OD_600_ at 48 h post-induction, and (2) the final OD_600_ of the culture (Additional file 1: Table S1). Both responses were fitted to polynomial models by least squares regression, and the coefficients of the models were hierarchically corrected and validated by analysis of variance (ANOVA).

To optimize both responses simultaneously, we employed the *desirability* function [28]. This procedure involves transformation of each response variable estimated by the RSM to an individual desirability function ${d}_{i}$, $0\le {d}_{i}\le 1$. If ${d}_{i}$ = 0, the response variable has a totally undesirable value, and it increases until ${d}_{i}$ = 1, where the response has a totally desirable one. In our case, to optimize the coculture production, both responses were to be maximized, so for each one, a one-sided transformation was applied as follows:

$${d}_{i}=\left\{\begin{array}{cc}0& {\widehat{Y}}_{i}\le {Y}_{i}^{min}\\ {\left(\frac{{\widehat{Y}}_{i}-{Y}_{i}^{min}}{{Y}_{i}^{max}-{Y}_{i}^{min}}\right)}^{r}& {Y}_{i}^{min}\le {\widehat{Y}}_{i}\le {Y}_{i}^{max}\\ 1& {\widehat{Y}}_{i}\ge {Y}_{i}^{max}\end{array}\right.$$

where ${\widehat{Y}}_{i}$ is the predicted response using the fitted model, ${Y}_{i}^{min}$ and ${Y}_{i}^{max}$ are the lowest and highest values obtained for the response $i$, respectively, and $r$ is a positive constant. For our analysis $r=1$, indicating that ${d}_{i}$ increases linearly as ${\widehat{Y}}_{i}$ increases.

The ${d}_{i}$ functions were then combined using the geometric mean to obtain a *global desirability function D,* that varies from 0 (value totally undesirable) to 1 (all responses are in a desirable range simultaneously) and can be defined as:

$$D={\left[\prod_{i=1}^{N}{\left({d}_{i}\right)}^{{w}_{i}}\right]}^{1/\sum_{i=1}^{N}{w}_{i}}$$

where ${w}_{i}$ is a weight that controls the relative importance assigned to each response $i$ in the expression of the function *D*. ${w}_{i}$ allows to include the researcher’s priorities and desires on building the optimization procedure. For our coculture, if ${d}_{1}$= desirability function for MBE production and ${d}_{2}$= desirability function for final OD_600_, we decided to give both responses equal importance, so ${w}_{1}$=1, ${w}_{2}$=1 and we get the simplified version $D={\left[\left({d}_{1}\right)\times \left({d}_{2}\right)\right]}^{1/2}$. Finally, this *D* function was maximized to find the best combination of the independent variables that optimized both responses simultaneously.

All the statistical analysis, model fitting, response surface plots and optimization were performed with the software package Design Expert ® v7 (Stat-Ease Inc, Minneapolis, USA). p-value < 0.05 was considered as significant. Interactive versions of the response surface plots were also built with Plotly’s Python library and can be found online at: https://github.com/ferbracalente/E.-coli-coculture.

### Fed-batch bioreactor cultivation

Fed-batch fermentations were carried out in a 2 L bioreactor BioFlo 115 (Eppendorf—New Brunswick Scientific), containing 1 L of M9 medium with the addition of 2 g of yeast extract, 20 g glucose, 0.5 g MgSO_4_.7H_2_O, 1 mL trace elements (50-mM FeCl_3_, 20-mM CaCl_2_, 10-mM ZnSO_4_, 10-mM MnCl_2_, 2-mM CoCl_2_, 2-mM CuCl_2_, 2-mM NiCl_2_, 2-mM H_3_BO_3_, 2-mM Na_2_MoO_4_, and 2-mM Na_2_SeO_3_) and the corresponding antibiotics. The dissolved oxygen (DO) probe and pH probe were calibrated according to the manufacturer’s protocol. Temperature, pH, DO, OD_600_ and glucose concentration were continuously measured along the process. The pH of the medium was maintained at 7.0 ± 0.1 by automatic addition of 15% NH_4_OH. Foam formation was controlled by the addition of Antifoam A (Sigma Aldrich) to the vessel. Glucose concentration was measured with an enzymatic kit (Wiener Lab, code 1,400,101). Agitation was set to a fixed value of 900 rpm, and oxygen was maintained above 20% of air saturation alternating air/O_2_ gas supply.

To inoculate the bioreactor, the constituent strains of the coculture RQ5.2/CB1.2 were grown separately in 10 mL M9 medium with 10 g/L glucose until exponential phase, and then mixed in the bioreactor. RQ5.2 strain was diluted to an OD_600_ of 0.001 and CB1.2 strain was diluted according to the indicated inoculation ratio. Temperature was maintained at 37 °C, and an initial batch phase of growth of  ~ 12 h was carried out until all glucose was consumed. Then, a nutrient solution consisting of 600 g/L glucose and 15 g/L MgSO_4_.7H_2_O was fed to the bioreactor at a constant rate of 0.23 mL/min until OD_600 _ ~ 75 was reached. At this moment, temperature was reduced to 23 °C and heterologous protein expression and MBE production were induced by the addition of IPTG 0.1 mM. Simultaneously, 2 mM sodium propionate and 20 µM biotin were also added to the culture, and the glucose flow was reduced to 0.08 mL/min to avoid its accumulation. 24 h later, a second pulse of 0.1 mM IPTG and 2 mM sodium propionate was added. Finally, the fed-batch phase was continued to conclude the process at  ~ 60 h of total duration. Samples were taken periodically for metabolite analysis and strain-to-strain ratio determination.

### Determination of strain-to-strain ratio of coculture

Real-time coculture population composition was determined by screening fluorescent/non-fluorescent colonies. The MBE producer strain (RQ5.2 strain) harbored pGFP, a Cm^R^ plasmid that express the green fluorescent protein (GFP) derived from the jellyfish *Aequorea Victoria*, while the BCA producer strain (CB1.2) was transformed with pBAD, a non-fluorescent Cm^R^ plasmid only to allow for antibiotics compatibility. To conduct the screening, serial dilutions of 100 µL of the coculture sample were made up to 10^8^-fold, and then grown for 24 h on LB agar plates containing Km, Cm and IPTG. The whole plates were screened with Olympus MVX10 MacroView fluorescence microscope, to identify and count the fluorescent and non-fluorescent colonies. The strain-to-strain ratio was calculated as (Fluorescent colonies)/(Total colonies) × 100, and expressed as %RQ5.2.

## Supplementary Information

**Additional file 1.** Additional tables and equations.

**Additional file 2.** Additional figures.

## Data Availability

The datasets used and/or analyzed during the current study and that are not included in this published article, are available from the corresponding author on reasonable request.

## Abbreviations

FA: Fatty acids
FAME: Fatty acid-methyl-esters
FAEE: Fatty acid-ethyl-esters
MA: Mycocerosic acid
MBFA: Multi-methyl-branched-fatty acids
MBE: Multi-methyl-branched esters
BCA: Branched-chain alcohols
RSM: Response surface methodology
OVAT: One variable at a time
CCD: Central composite design
