# Supplementary material for: Escherichia coli coculture for de novo production of esters derived of methyl-branched alcohols and multi-methyl branched fatty acids
Source: Microb Cell Fact. 2022 Jan 15;21:10. doi: 10.1186/s12934-022-01737-0 (PMC8760833; doi:10.1186/s12934-022-01737-0)
Supplement: Supplementary file 1 — Additional file 1. Additional tables and equations. [file 12934_2022_1737_MOESM1_ESM.docx]

**Table S1.** Central composite design (CCD) used for the optimization of the responses.

| Experiments | Blocks | Factors |  |  |  | Responses |  |
| --- | --- | --- | --- | --- | --- | --- | --- |
|  |  | Inoculation ratio  (% RQ5.1) | Time (h) | IPTG (mM) |  | BCA-MBE  production (A.U.) | Final OD_600_ |
| 1 | Day1 | 50.0 | 2.5 | 0.23 |  | 21.9 | 9.11 |
| 2 | Day1 | 74.3 | 1.3 | 0.33 |  | 16.6 | 7.30 |
| 3 | Day1 | 25.7 | 1.3 | 0.12 |  | 12.2 | 9.21 |
| 4 | Day1 | 25.7 | 3.7 | 0.12 |  | 3.4 | 8.92 |
| 5 | Day1 | 74.3 | 1.3 | 0.12 |  | 5.1 | 8.98 |
| 6 | Day1 | 74.3 | 3.7 | 0.33 |  | 0.0 | 7.42 |
| 7 | Day1 | 74.3 | 1.3 | 0.33 |  | 14.9 | 7.35 |
| 8 | Day1 | 74.3 | 3.7 | 0.33 |  | 0.0 | 7.68 |
| 9 | Day1 | 25.7 | 1.3 | 0.33 |  | 9.4 | 6.52 |
| 10 | Day1 | 25.7 | 1.3 | 0.33 |  | 10.0 | 6.44 |
| 11 | Day1 | 25.7 | 3.7 | 0.33 |  | 18.1 | 8.13 |
| 12 | Day1 | 50.0 | 2.5 | 0.23 |  | 23.0 | 9.18 |
| 13 | Day1 | 74.3 | 1.3 | 0.12 |  | 5.6 | 8.77 |
| 14 | Day1 | 25.7 | 3.7 | 0.33 |  | 14.8 | 8.45 |
| 15 | Day1 | 74.3 | 3.7 | 0.12 |  | 0.1 | 9.92 |
| 16 | Day1 | 74.3 | 3.7 | 0.12 |  | 0.1 | 9.62 |
| 17 | Day1 | 50.0 | 2.5 | 0.23 |  | 22.9 | 9.17 |
| 18 | Day1 | 50.0 | 2.5 | 0.23 |  | 21.6 | 9.97 |
| 19 | Day1 | 25.7 | 1.3 | 0.12 |  | 12.2 | 8.79 |
| 20 | Day1 | 25.7 | 3.7 | 0.12 |  | 2.7 | 8.98 |
| 21 | Day2 | 90.9 | 2.5 | 0.23 |  | 3.6 | 7.29 |
| 22 | Day2 | 50.0 | 2.5 | 0.23 |  | 25.1 | 6.84 |
| 23 | Day2 | 50.0 | 4.5 | 0.23 |  | 4.8 | 6.20 |
| 24 | Day2 | 50.0 | 2.5 | 0.05 |  | 0.0 | 7.53 |
| 25 | Day2 | 50.0 | 2.5 | 0.40 |  | 16.6 | 5.02 |
| 26 | Day2 | 50.0 | 2.5 | 0.40 |  | 19.0 | 5.73 |
| 27 | Day2 | 9.1 | 2.5 | 0.23 |  | 9.5 | 3.80 |
| 28 | Day2 | 50.0 | 2.5 | 0.23 |  | 27.6 | 7.19 |
| 29 | Day2 | 50.0 | 4.5 | 0.23 |  | 14.6 | 7.62 |
| 30 | Day2 | 90.9 | 2.5 | 0.23 |  | 5.1 | 7.61 |
| 31 | Day2 | 50.0 | 2.5 | 0.05 |  | 0.0 | 7.29 |
| 32 | Day2 | 9.1 | 2.5 | 0.23 |  | 15.8 | 4.29 |
| 33 | Day2 | 50.0 | 0.5 | 0.23 |  | 15.0 | 5.99 |
| 34 | Day2 | 50.0 | 0.5 | 0.23 |  | 13.2 | 4.51 |

Highlighted rows correspond to the six replicates for the center point of the design. BCA-MBE, BCA-derived MBE; A.U., arbitrary units from densitometric quantification of MBE determined by TLC analysis.

**Table S2.** Analysis of variance (ANOVA) for Response Surface Reduced Cubic Model for BCA-derived MBE production

| Source | Sum of squares | DF | Mean square | *F* value | *p*-value |
| --- | --- | --- | --- | --- | --- |
| Block | 16.41 | 1 | 16.41 |  |  |
| Model | 2188.26 | 12 | 182.36 | 38.28 | < 0.0001 |
| A- %RQ5.1 | 170.22 | 1 | 170.22 | 35.73 | < 0.0001 |
| B- Time (h) | 19.61 | 1 | 19.61 | 4.12 | 0.0560 |
| C- IPTG (mM) | 8.11 | 1 | 8.11 | 1.70 | 0.2069 |
| AB | 86.59 | 1 | 86.59 | 18.18 | 0.0004 |
| AC | 0.097 | 1 | 0.097 | 0.020 | 0.8878 |
| BC | 7.32 | 1 | 7.32 | 1.54 | 0.2293 |
| A^2^ | 711.56 | 1 | 711.56 | 149.38 | < 0.0001 |
| B^2^ | 440.63 | 1 | 440.63 | 92.50 | < 0.0001 |
| C^2^ | 676.74 | 1 | 676.74 | 142.07 | < 0.0001 |
| ABC | 173.91 | 1 | 173.91 | 36.51 | < 0.0001 |
| A^2^B | 17.05 | 1 | 17.05 | 3.58 | 0.0731 |
| C^3^ | 46.58 | 1 | 46.58 | 9.78 | 0.0053 |
| Residual | 95.27 | 20 | 4.76 |  |  |
| Lack of fit | 9.70 | 2 | 4.85 | 1.02 | 0.3804 |
| Pure error | 85.57 | 18 | 4.75 |  |  |

**Table S3.**Coded values of the independent variables used in the central composite design.

| Variable | Component | Level | | | | |
| --- | --- | --- | --- | --- | --- | --- |
|  | | -2 | -1 | 0 | 1 | 2 |
| A | % RQ5.1 at inoculation | 9 | 25 | 50 | 75 | 91 |
| B | Induction time (h) | 0.5 | 1.3 | 2.5 | 3.7 | 4.5 |
| C | IPTG (mM) | 0.05 | 0.12 | 0.23 | 0.33 | 0.4 |

**Eq. S1**. Reduced cubic model for the final OD_600_ of the coculture in terms of the coded values of the independent variables A, B and C.

$$Y_{2}= 8.20+1.01 A+0.41B-0.76C-0.079 AB-0.074 AC+0.15 BC-0.47 A^{2}-0.35 B^{2}-0.24 C^{2}-0.32 ABC-0.91 {AB}^{2}$$

$Y_{2}$, final OD_600_

*A,* inoculation ratio (%RQ5.1)

*B*, inoculation time (h)

*C*, inducer concentration.

**Table S4.** Analysis of variance (ANOVA) for Response Surface Reduced Cubic Model for final OD_600_

| Source | Sum of squares | DF | Mean square | *F* value | *p*-value |
| --- | --- | --- | --- | --- | --- |
| Block | 43.10 | 1 | 43.10 |  |  |
| Model | 40.39 | 11 | 3.67 | 19.53 | < 0.0001 |
| A- %RQ5.1 | 11.58 | 1 | 11.58 | 61.56 | < 0.0001 |
| B- Time (h) | 4.69 | 1 | 4.69 | 24.93 | < 0.0001 |
| C- IPTG (mM) | 15.75 | 1 | 15.75 | 83.76 | < 0.0001 |
| AB | 0.099 | 1 | 0.099 | 0.53 | 0.4756 |
| AC | 0.087 | 1 | 0.087 | 0.46 | 0.5038 |
| BC | 0.35 | 1 | 0.35 | 1.87 | 0.1863 |
| A^2^ | 4.91 | 1 | 4.91 | 26.09 | < 0.0001 |
| B^2^ | 2.74 | 1 | 2.74 | 14.56 | 0.0010 |
| C^2^ | 1.27 | 1 | 1.27 | 6.77 | 0.0167 |
| ABC | 1.60 | 1 | 1.60 | 8.51 | 0.0082 |
| AB^2^ | 5.49 | 1 | 5.49 | 29.20 | < 0.0001 |
| Residual | 3.95 | 21 | 0.19 |  |  |
| Lack of fit | 0.58 | 3 | 0.19 | 1.03 | 0.4028 |
| Pure error | 3.37 | 18 | 0.19 |  |  |

**Table S5.**Primers used in this study.

| Primer name | Sequence | Reference |
| --- | --- | --- |
| pBAD_Cm_F | ATTATGCATATCACTTATTCAGGCGTAGC | This work |
| pBAD_Cm_R | ACTATGCATTGCGCCGAATAAATACCTG | This work |
| kivD_Fw | AGCATATGTATACAGTAGGAGATTACCTAT | This work |
| kivD_Rv | AGACTAGTTTATGATTTATTTTGTTCAGCAAAT | This work |
| ADH2_Fw | AGCATATGTCTATTCCAGAAACTCAAAAAG | This work |
| ADH2_Rv | AGACTAGTTTATTTAGAAGTGTCAACAACGTAT | This work |
| P1 | CTGCTTGTGGTGGTGAAT | [1] |
| P2 | ACTTAACGGCTGACATGG | [1] |
| P3 | ACGAGTATCGAGATGGCA | [1] |
| P4 | TAAGGCAAGACGATCAGG | [1] |

**Additional file 1 references**

1. Haldimann A, Wanner BL. Conditional-replication, integration, excision, and retrieval plasmid-host systems for gene structure-function studies of bacteria. Journal of Bacteriology. 2001;183:6384–93.
