# Supplementary material for: Escherichia coli coculture for de novo production of esters derived of methyl-branched alcohols and multi-methyl branched fatty acids
Source: Microb Cell Fact. 2022 Jan 15;21:10. doi: 10.1186/s12934-022-01737-0 (PMC8760833; doi:10.1186/s12934-022-01737-0)
Supplement: Supplementary file 2 — Additional file 2. Additional figures. [file 12934_2022_1737_MOESM2_ESM.docx]

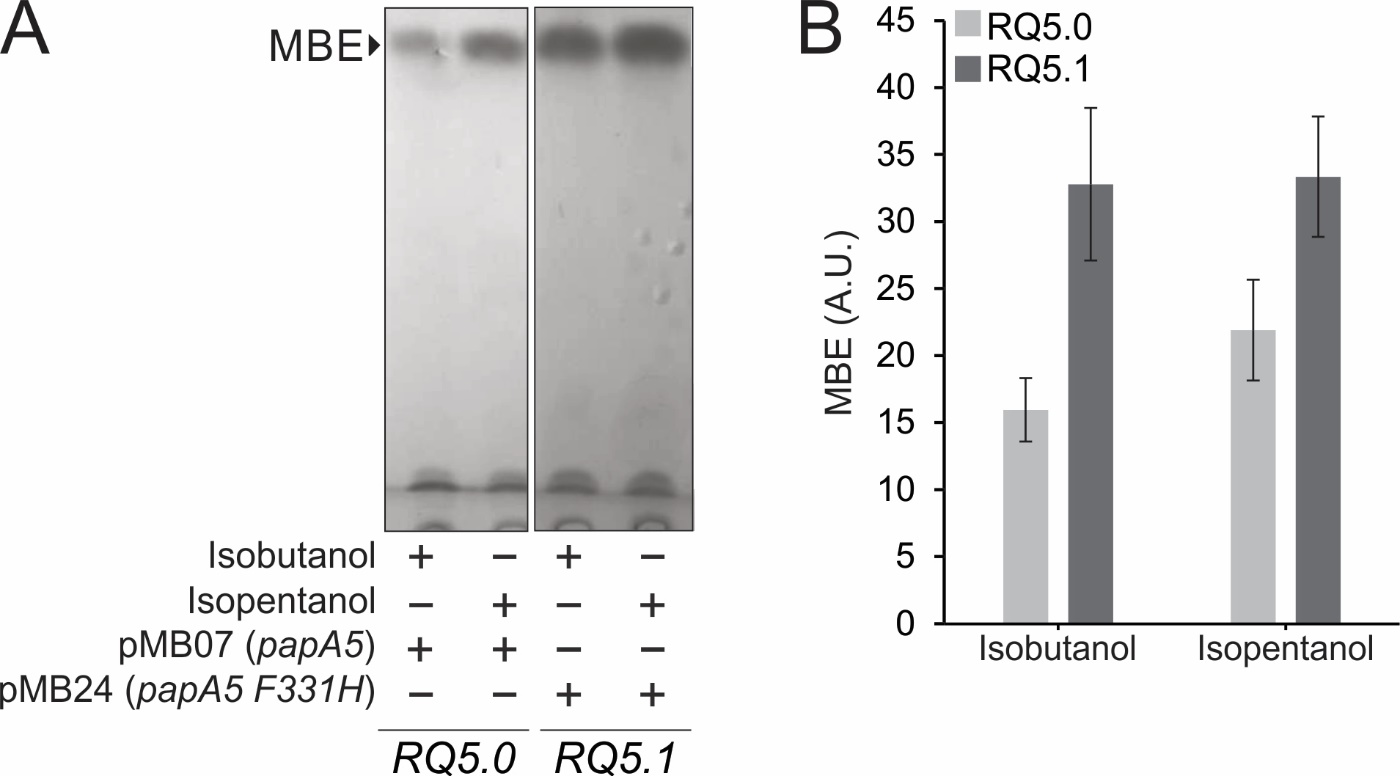


**Fig. S1. Mutant PapA5 F331H increases BCA-derived MBE titers in the RQ5 strain.** (A) TLC showing BCA-derived MBE production 48 hours post induction of RQ5.0 (RQ5/pMB07) and RQ5.1 (RQ5/pMB24) strains with 10mM isobutanol and isopentanol supplemented to the medium. (B) Densitometric measurements of the TLC spots corresponding to isobutanol and isopentanol-derived MBE for both RQ5.0 and RQ5.1 strains. Error bars represent the standard deviation of three biological replicates. A.U., arbitrary units.

**
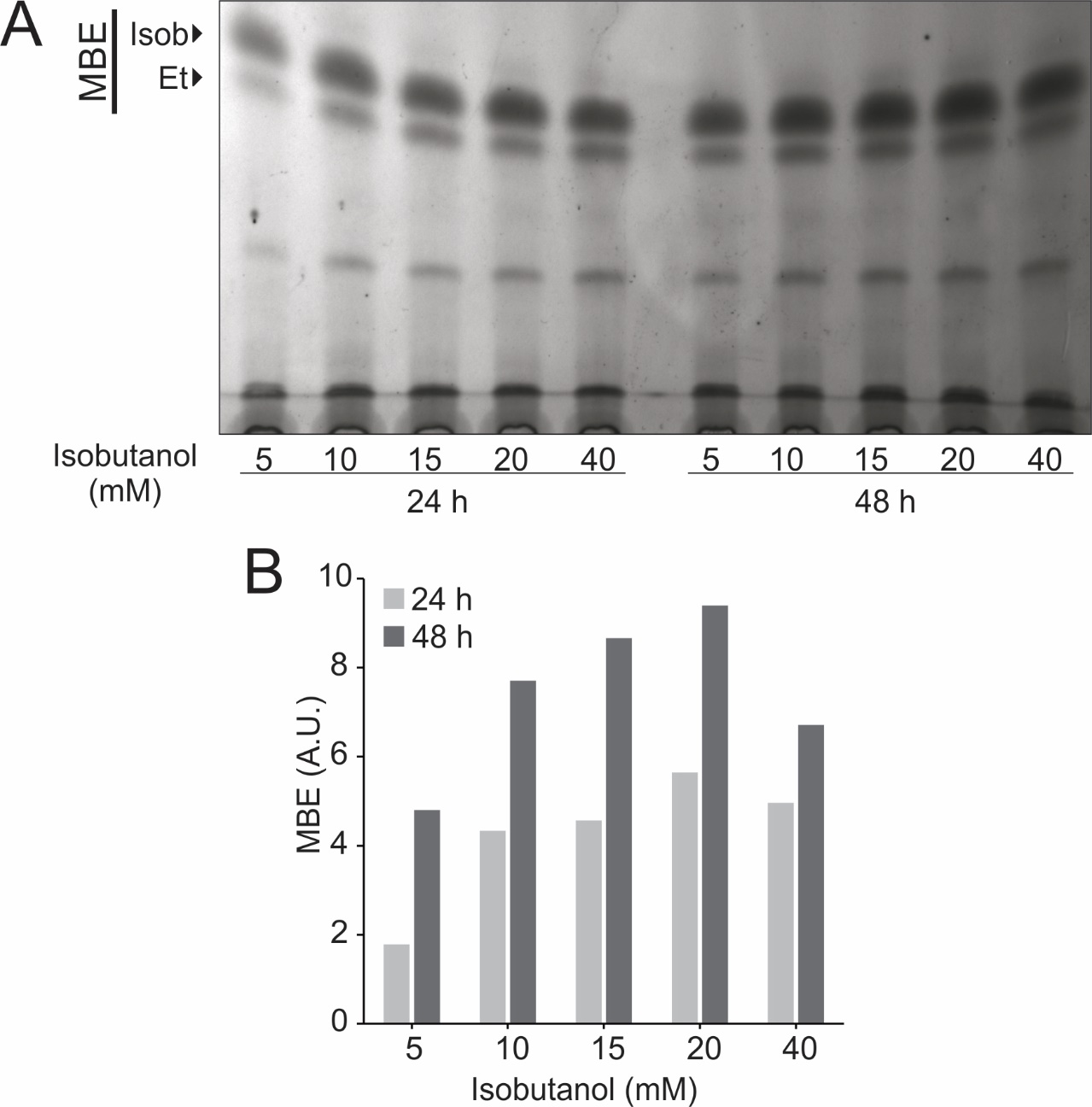
**

**Fig. S2. MBE production depends on the external alcohol concentration.** (A) TLC showing the total lipid pattern and MBE production 24 and 48 hours post induction of RQ5.1 strain with isobutanol supplemented to the culture medium at the final concentration indicated at the bottom of the TLC. (B) Densitometric measurements of the TLC spots corresponding to isobutanol-derived MBE were performed using ImageJ software. Et, ethanol derived MBE; Isob, isobutanol derived MBE; A.U., arbitrary units.

**
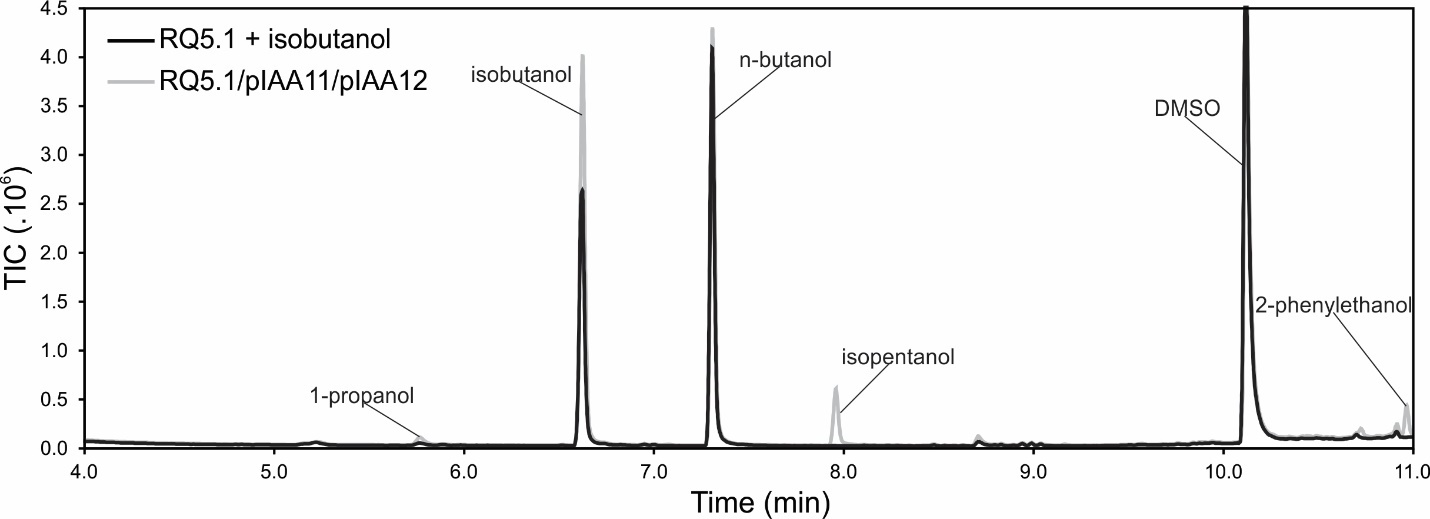
**

**Fig. S3. Analysis of alcohol production by RQ5.1/pIAA11/pIAA12 strain.** GC-MS chromatograms of supernatant samples 48 hours post induction of RQ5.1 supplemented with isobutanol and RQ5.1/pIAA11/pIAA12 strains. 2-phenylethanol and isopentanol arise as differential compounds. n-butanol diluted in DMSO was added to the samples as internal standard. 1-propanol at very low levels can also be detected in both samples. TIC, total ion current.


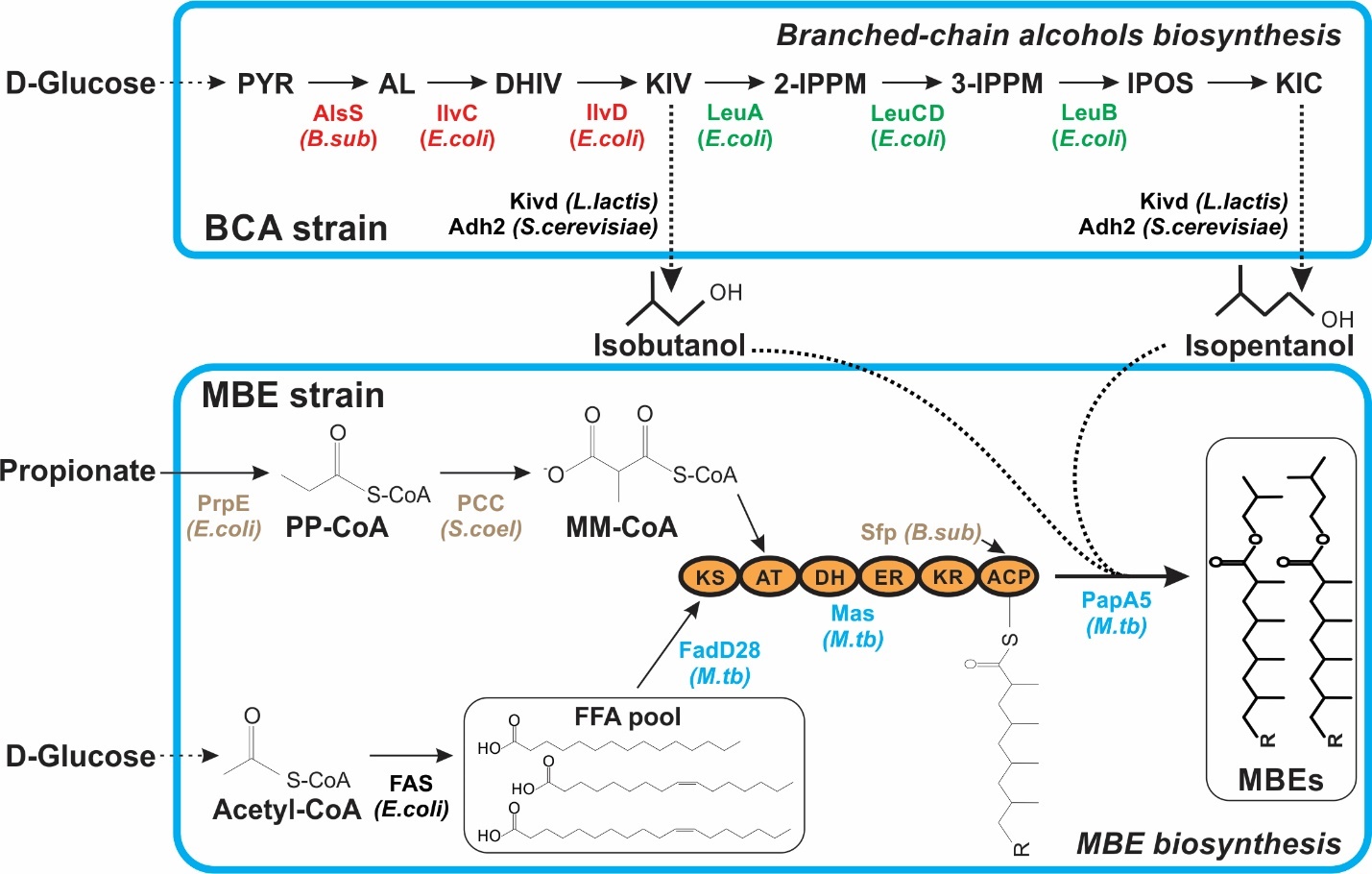


**Fig. S4. Division of the BCA-derived MBE production pathway into two strains.** Representation of the constituent strains of the coculture, one containing the branched-chain alcohols biosynthesis pathway (BCA strain) and the other containing the MBE biosynthesis pathway (MBE strain). Enzymes highlighted in red are introduced with plasmid pIAA11, the ones highlighted in green with plasmid pIAA15, the ones in light blue with plasmid pMB24, and those in brown are integrated into the chromosome and under the control of T7 promoters.

**
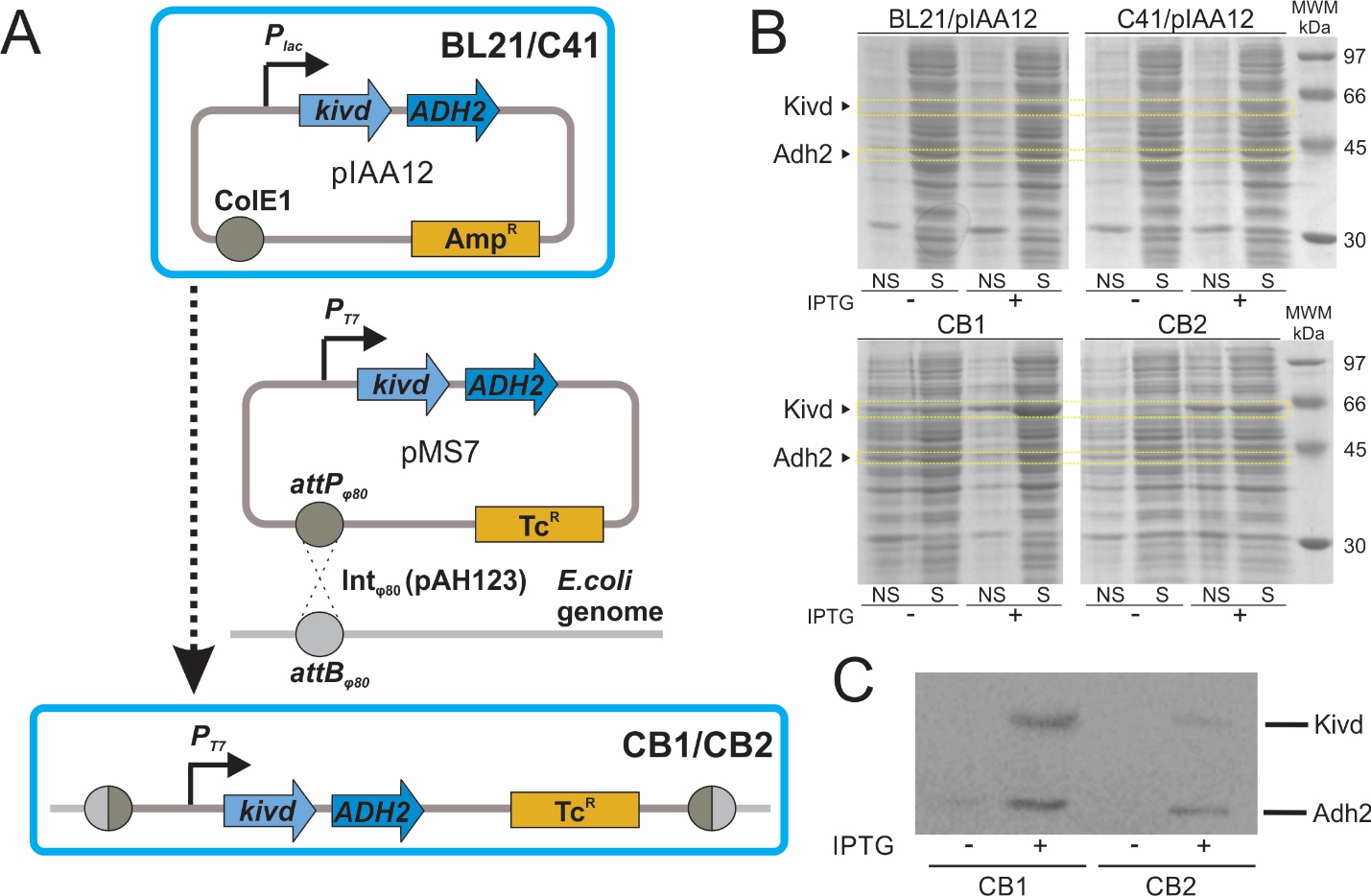
**

**Fig. S5. Redesign of the BCA producing strains by overexpression of Kivd and Adh2.** (A) *kivd* and *ADH2* genes were cloned as an operon under the strong T7 promoter (*P_T7_*) in the integrative plasmid pMS7. pMS7 was transformed into BL21 and C41 strains, harboring the helper plasmid pAH123 that expresses Int_φ80,_ and integrated into their genomes at the *attB _φ80_* site, yielding CB1 and CB2 strains, respectively. (B) Coomasie-stained SDS-PAGE of total protein extracts to compare Kivd and Adh2 expression levels from plasmid (BL21/pIAA12 and C41/pIAA12) and after integration in the chromosome (CB1 and CB2). (-), non-induced; (+) induced with IPTG; NS, non-soluble fraction; S, soluble fraction; MWM, molecular weight marker. (C) Western blot of total protein extracts from CB1 and CB2 strains, incubated with monoclonal anti-6xHis antibody. Correct expression of both Kivd and Adh2 enzymes was confirmed, with higher levels in CB1 strain. (-) non-induced; (+) induced with IPTG.

**
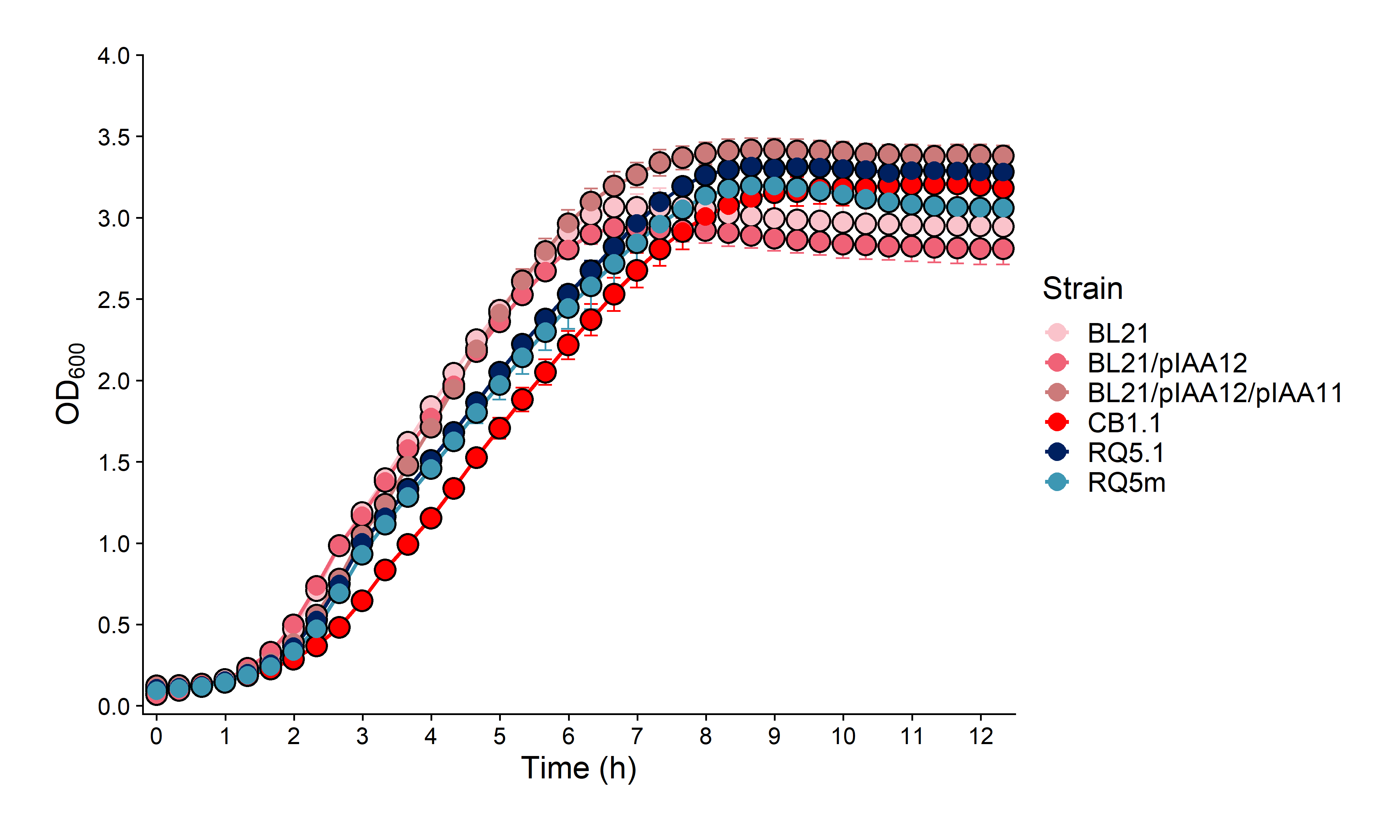
Fig. S6. Growth curves of BCA and BCA-derived MBE single producer strains.** Growth curves at 37° C in M9 medium with 1% glucose in a 96-well microplate at an initial OD_600_ = 0.02. Error bars represent the standard deviation from three biological replicates. OD_600_ = optical density measured at 600 nm.

**
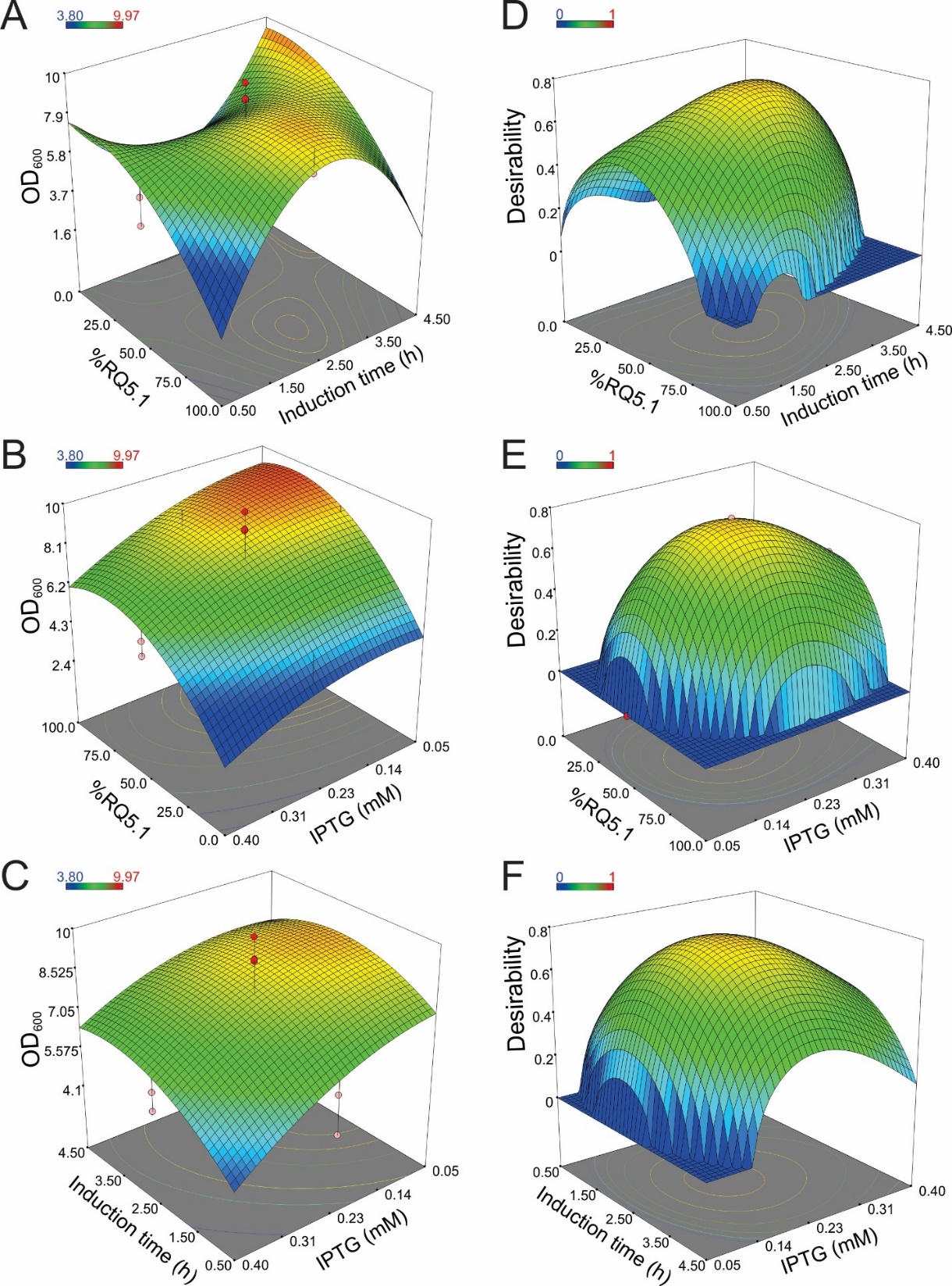
**

**Fig. S7. Simultaneous optimization of the coculture.** (A-C) Response surface plots for the final OD_600nm_ (OD_600_) of the coculture as a function of the variables: inoculation ratio (% RQ5.1), induction time (h), and inducer (IPTG) concentration (mM). These plots were obtained for a given pair of variables, while maintaining the other one fixed at its zero value. (D-F) Response surface plots for the global desirability function *D* as a function of the three variables under study. These plots were obtained for a given pair of variables, while maintaining the other one fixed at its optimum value. Contour plots are projected on the *x*-*y* plane. The red and pink points correspond to experimental design points. Interactive versions of the response surface plots at: <https://github.com/ferbracalente/E.-coli-coculture>.


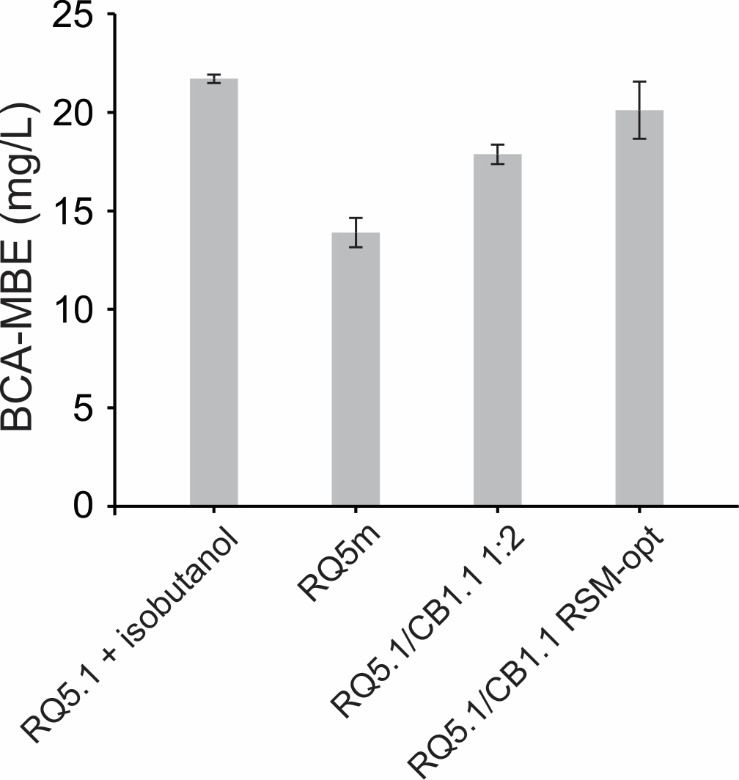


**Fig. S8. Comparison of BCA-derived MBE production titers.** BCA-derived MBE production for the RQ5.1 strain with 20mM isobutanol added to the culture medium (RQ5.1 + isobutanol) compared with the *de novo* production strategies: RQ5m monoculture, RQ5.1/CB1.1 coculture (RQ5.1/CB1.1 1:2) and RQ5.1/CB1.1 coculture after RSM optimization (RSM-opt).





**Fig. S9. Different growth rates for the constituent strains of the coculture in batch cultures.**  (A) and (B). Growth curves of RQ5.2 and CB1.2 strains at 37° C in M9 medium with 2% glucose, at two different inoculation dilutions (initial OD_600_ 0.02 and 0.001), performed in a 96-well microplate (A) and in shake flasks (B). (C) Growth curves of RQ5.2 and CB1.2 strains in 1 L of M9 medium with 20 g/L glucose, performed at 37° C in a 2 L bioreactor. Error bars represent the standard deviation from three biological replicates. OD_600_ = optical density measured at 600 nm.
